# Transcriptome Profiling Reveals New Insights into the Immune Microenvironment and Upregulation of Novel Biomarkers in Metastatic Uveal Melanoma

**DOI:** 10.3390/cancers12102832

**Published:** 2020-09-30

**Authors:** Yamini Krishna, Amelia Acha-Sagredo, Dorota Sabat-Pośpiech, Natalie Kipling, Kim Clarke, Carlos R. Figueiredo, Helen Kalirai, Sarah E. Coupland

**Affiliations:** 1 Liverpool Clinical Laboratories, Liverpool University Hospitals NHS Foundation Trust, Duncan Building, Daulby Street, Liverpool L69 3GA, UK; ykrishna02@hotmail.com; 2 Liverpool Ocular Oncology Research Centre, Department of Molecular and Clinical Cancer Medicine, University of Liverpool, William Henry Duncan Building, West Derby Street, Liverpool L7 8TX, UK; amelia.acha-sagredo@kcl.ac.uk (A.A.-S.); dorota.sabat@liverpool.ac.uk (D.S.-P.); natalie.kipling@liverpool.ac.uk (N.K.); h.kalirai@liverpool.ac.uk (H.K.); 3 Computational Biology Facility, Biosciences Building, University of Liverpool, Crown Street, Liverpool L69 7ZB, UK; kim.clarke@liverpool.ac.uk; 4 MediCity Research Laboratory and Institute of Biomedicine, University of Turku, Turun yliopisto, FI-20014 Turku, Finland; carlosroger20@gmail.com

**Keywords:** metastatic uveal melanoma, transcriptome profiling, immunotherapy, MUM1, DUSP4

## Abstract

**Simple Summary:**

Uveal melanoma (UM) is a rare aggressive eye cancer. Although treatment of the eye tumour is successful, about 50% of UM patients develop a relapse of their cancer in the liver. At present, such advanced disease is not curable. A better understanding of the metastatic UM (mUM) in the liver is essential to improve patient survival. This study examines both the response of immune cells within the liver to the UM secondaries (metastases), as well as the expression of various proteins by the UM cells. Our study demonstrates that there is a limited immune response to the mUM, but reveals that a certain type of reactive immune cell: a protumourigenic subset of macrophage is dominant within the mUM. Our research also reveals novel proteins within the mUM, which are specific to these cells and therefore may be targetable in future therapies.

**Abstract:**

Metastatic uveal melanoma (mUM) to the liver is incurable. Transcriptome profiling of 40 formalin-fixed paraffin-embedded mUM liver resections and 6 control liver specimens was undertaken. mUMs were assessed for morphology, nuclear BAP1 (nBAP1) expression, and their tumour microenvironments (TME) using an “immunoscore” (absent/altered/high) for tumour-infiltrating lymphocytes (TILs) and macrophages (TAMs). Transcriptomes were compared between mUM and control liver; intersegmental and intratumoural analyses were also undertaken. Most mUM were epithelioid cell-type (75%), amelanotic (55%), and nBAP1-ve (70%). They had intermediate (68%) or absent (15%) immunoscores for TILs and intermediate (53%) or high (45%) immunoscores for TAMs. M2-TAMs were dominant in the mUM-TME, with upregulated expression of *ANXA1*, *CD74*, *CXCR4*, *MIF*, *STAT3*, *PLA2G6*, and *TGFB1*. Compared to control liver, mUM showed significant (*p* < 0.01) upregulation of 10 genes: *DUSP4*, *PRAME*, *CD44*, *IRF4/MUM1*, *BCL2*, *CD146/MCAM/MUC18*, *IGF1R*, *PNMA1*, *MFGE8/lactadherin*, and *LGALS3/Galectin-3*. Protein expression of DUSP4, CD44, IRF4, BCL-2, CD146, and IGF1R was validated in all mUMs, whereas protein expression of PRAME was validated in 10% cases; LGALS3 stained TAMs, and MFGEF8 highlighted bile ducts only. Intersegmental mUMs show differing transcriptomes, whereas those within a single mUM were similar. Our results show that M2-TAMs dominate mUM-TME with upregulation of genes contributing to immunosuppression. mUM significantly overexpress genes with targetable signalling pathways, and yet these may differ between intersegmental lesions.

## 1. Introduction

Uveal melanoma (UM) is an aggressive primary intraocular malignancy with ~50% of UM patients developing metastatic disease, usually in the liver, during the first decade after primary treatment [1,2,3,4,5]. UM is distinct from skin melanoma and characterized by certain genetic alterations that are unique to this tumour [1,2,6,7,8,9,10]. Strong genetic prognosticators for increased metastatic risk in UM include monosomy 3 (M3), polysomy 8q, inactivating somatic mutations of *BAP1*, and somatic mutations in *SF3B1/SRSF2* [2,6,9,10,11,12,13,14,15,16,17,18,19].

Current treatments for metastatic UM (mUM) to the liver include metastasectomy, liver lobe resection, systemic chemotherapy, or isolated liver perfusion, as well as radiofrequency ablation. However, none of these therapies are curative [2,4,20,21]. Consequently, there is an urgent need to improve current treatments for established mUM and to develop neoadjuvant therapies when mUM tumour volumes are low or even clinically imperceptible [22].

In contrast to metastatic skin melanoma where immunotherapy using immune check point inhibitors (ICIs) has significantly improved patient outcomes [23], ICIs in mUM have demonstrated only marginal success [24]. Intriguingly, UM has relatively low immunogenicity in both the eye and its metastatic sites. The underlying characteristics of the tumour microenvironment (TME) of mUM resulting in its ability for “immune escape” are poorly understood, but have become the subject of intensive investigation [25,26].

Our previous morphological analyses demonstrated that the main reactive cells within mUM are CD163+ tumour-associated macrophages (TAMs) [25], i.e., protumourigenic M2 phenotype. Any tumour infiltrating lymphocytes (TILs) present in mUM were CD8+ T-cells with low expression of cytotoxic markers, such as Granzyme B, and they were typically “excluded” from the tumour core [25]. These results, which were supported by others and suggest CD8+ T-cell exhaustion [27,28], were corroborated by our earlier study using NanoString analyses of six hepatic mUM by Figueiredo et al. 2020 [26]. We showed that within mUM, conventional immune checkpoint regulators (CTLA-4, PD1 and PD-L1) were low and that immune suppressive genes (e.g., *HLA-DRA*, *CD38*, *CD74*, *LGALS3*) were increased [26].

Herein, we build upon our preliminary findings by undertaking a NanoString-based transcriptome analysis of a larger and separate cohort of hepatic mUM, examining not only the gene expression profile of the mUM but also the TME in and around the lesions. We reveal the expression of novel markers, which are specific to mUM (i.e., not expressed by adjacent hepatic tissue) and thereby may be the focus of targeted therapy.

## 2. Results

### 2.1. Patients and Samples

Detailed clinical, pathological, and genetic data for a smaller cohort of the mUM cases have been previously published by our group [25,29]. For the current study, a total of 40 resected hepatic mUM from 27 patients (Table S1) and six control liver specimens were included. The cases were from 15 men and 12 women, with a median age of 54 years (range 32–78 years) at primary management (Table S1).

Morphologically, the mUM showed a nodular growth pattern in 31/40 (77.5%) samples, with 21 (52%) demonstrating complete fibrous encapsulation. The mUMs were of epithelioid cell type in 30/40 (75%), and the degree of pigmentation ranged from amelanotic in 22/40 (55%), i.e., partial in 11 (27.5%), moderate in three (7.5%), and heavy in four tumours (10%). Fourteen of the 40 (35%) mUM had necrotic foci of varying sizes. The majority of mUM (72.5%) were nBAP1-ve; however, 11/40 (27.5%) mUM showed clear nuclear expression for this marker (Table S1). Interestingly, one patient (R4, R34, and R35) had two metastasectomies, 6 years apart—with the first lesion (R4) demonstrating nBAP1- and the second scattered across two lobes (R34 and 35) being nBAP1+.

With respect to the immunoscore, most mUMs showed either “low” (6/40; 15%) or “intermediate” (27/40; 67.5%) scores for the TILs, whereas they demonstrated either “intermediate” (18/40; 45%) or “high” (21/40; 52.5%) scores for the TAMs. Interestingly, 23/27 mUMs with TILs of “intermediate” scores displayed the “altered excluded” pattern.

### 2.2. Transcriptomic Analyses of mUM Samples

**Figure 1:**
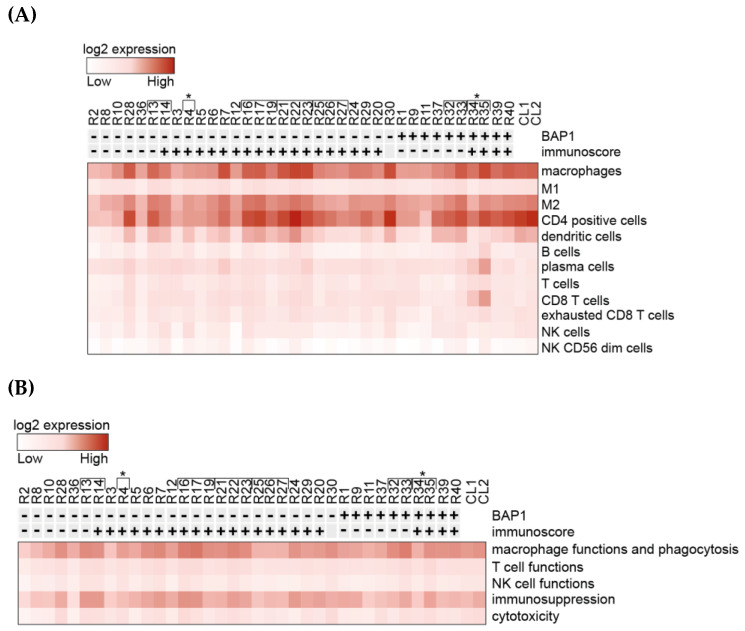
Heatmaps generated from normalized data post-NanoString analysis of gene expression. All metastatic uveal melanoma (mUM) cases were sorted by nuclear BAP1(nBAP1) status and total immunoscore (previously described, where total immunoscore of “0–3” i.e., “cold” tumours are shown as “-” and “4–6” i.e., “hot” tumours are marked “+.”) (**A**) Analysis of the relative participation of immune cell types/cell type profiling within the mUM tumour microenvironment. (**B**) Analysis of the gene profiles characteristic for the indicated cell functions. Key: [ indicates tumours from the same patient. *R4, R34, and R35 were samples from the same patient, with a 6-year difference between the two metastasectomies. CL1 is control liver samples pool 1 and CL2 is control liver samples pool 2. (Lists of genes included in each category for (**A**) and (**B**) are available in Figure S3A,B, respectively (as defined by NanoString, Figueiredo et al. [26] and Waks et al. [31])).

The dominant cell type expressed within the mUM was M2 macrophages (Figure 1A). Some genes associated with M2 macrophages were highly upregulated in mUM when compared to control liver and included: *ANXA1* (Annexin A1), *CD74/NT5E* (ecto-5′-nucleotidase), *CXCR4* (C-X-C motif chemokine receptor-4), *MIF* (macrophage migration inhibitory factor), *STAT3* (signal transducer and activator of transcription-3), *PLA2G6* (phospholipase A2 group-6), and *TGFB1* (transforming growth factor beta-1) (Figure S1).

CD4+ cell-related genes were also highly expressed; however, taking into consideration that the T-cell genes showed comparatively low expression (Figure 1A), the elevated levels of CD4 were attributed to its expression by macrophages [30]. Genes related to dendritic cells (DCs), plasma cells, and exhausted T-cells were the next most highly expressed markers (Figure 1A). When analysing gene profiles according to cell functions, the most highly expressed were those related to macrophage function and phagocytosis, as well as immunosuppression (Figure 1B). Of the immune checkpoint regulators (ICR), the majority of these genes showed low expression (e.g., *CTLA4* and *PDCD1*). Interestingly, however, CD276/*B7-H3*, *HMGB1* (high mobility group box 1), and CD73/*NT5E* were found to be highly expressed in both mUM and control liver specimens (Figure S2B).

**Figure 2:**
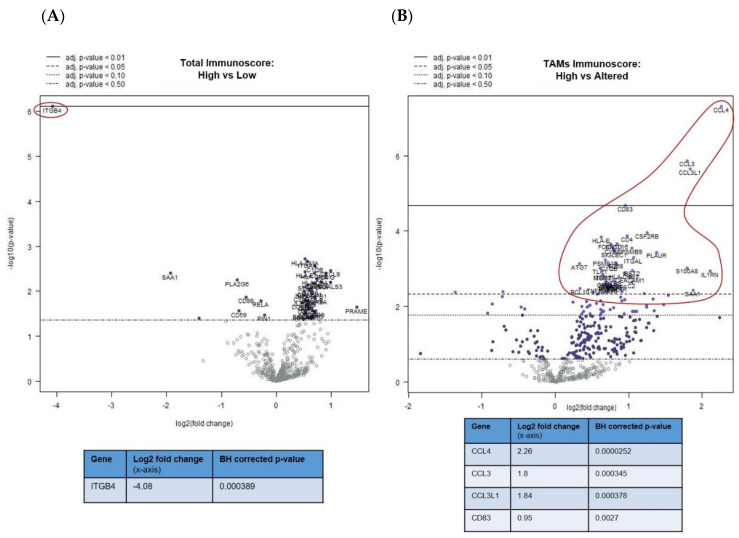

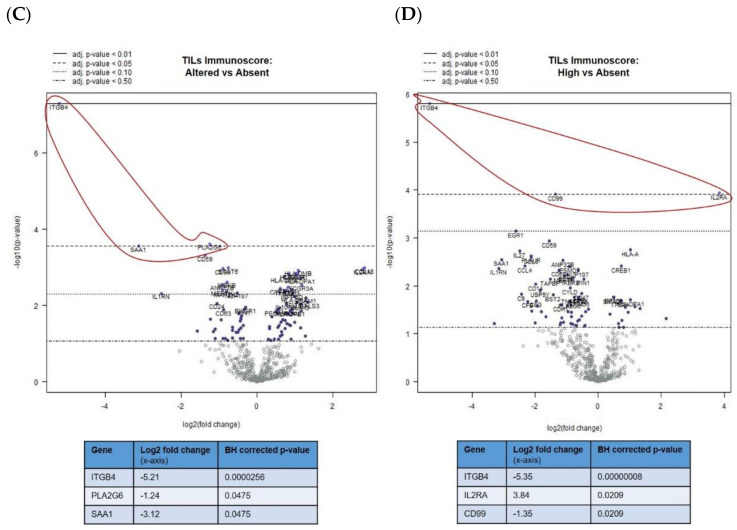
Differential gene expression profiles presented as volcano plots for: (**A**) total immunoscore of “high” versus “low” for all mUM, as previously defined, (**B**) tumour-associated macrophages (TAMs) immunoscore for all mUM illustrating “high” versus “altered,” the tumour infiltrating lymphocytes (TILs) immunoscore for all mUM showing (**C**) “altered/intermediate” versus “absent/cold,” and (**D**) “high/hot” versus “absent/cold.” For each volcano plot, the log_2_ fold changes with cutoffs for adjusted *p*-values are shown. The genes of interest (marked fold changes and statistically significant) are highlighted (red outline and inset table). (Table S2A–D gives more detailed output for each gene).

The differential expression (DE) analysis of the mUM partitioned on the basis of their total immunoscores showed a range of gene alterations in immune “hot” mUM relative to their immune “cold” counterparts. For instance, *ITGB4 (integrin ß4*; *CD104)* expression was 16 times lower (log_2_ fold change: 4) (Figure 2A) in mUM with high total immunoscores compared to those with low immunoscores (adjusted *p* < 0.01). Upregulation of gene markers of cytotoxic cells, macrophages, DCs, and exhausted CD8+ T-cells was observed, although they did not reach statistical significance (Table S2A). When comparing the DE of the mUM on the basis of their macrophage score (high versus altered), those mUM with high scores showed upregulation of chemokine ligands, e.g., *CCL4* (also known as macrophage inflammatory protein (MIP)-1β) (adjusted *p* < 0.01), *CCL3* (also known as MIP-1-α) (adjusted *p* < 0.01), *CCL3L1* (adjusted *p* < 0.01), as well as the activation marker *CD83* (adjusted *p* < 0.01) (Figure 2B). Genes for antigen processing, transporter functions, complement and other cytokines were also significantly upregulated but <2 log2 fold change (Table S2B).

The abovementioned decrease in *ITGB4* expression was also observed in the comparison of mUM with “altered versus absent” TIL scores (adjusted *p* < 0.01) (Figure 2C) and “high versus absent” TIL scores (adjusted *p* < 0.01) (Figure 2D). Other genes that were downregulated in the TIL “high” mUM were *PLA2G6*, *SAA1*, and *CD99* (adjusted *p* < 0.05 for all), whereas *CD25*/*IL2RA* (Treg marker) was upregulated (adjusted *p* < 0.05) (Figure 2C,D and Table S2C,D).

**Figure 3:**
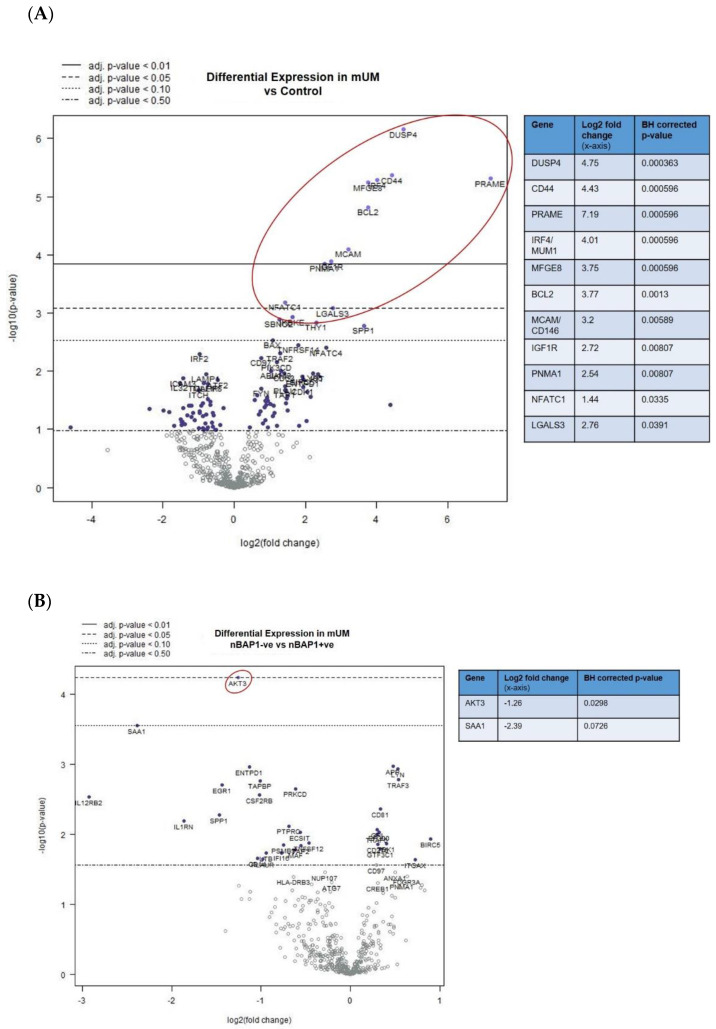
Differential gene expression profiles presented as volcano plots for: (**A**) all mUM versus control liver and (**B**) nBAP+ve mUM versus nBAP-ve mUM. The log^2^ fold changes with cut-offs for adjusted *p*-values are shown. The genes of interest (marked fold changes and statistically significant) are highlighted (red outline and inset table). (Table S3 gives more detailed output for each gene).

When undertaking DE analysis comparing the mUM with control liver, 11 genes had significantly increased expression (adjusted *p* < 0.01), ten of which had a log_2_ fold-change greater than 2. (Figure 3A; Table S3A). These included: *DUSP4* (dual specificity phosphatase 4), *PRAME* (Preferentially Expressed Antigen in Melanoma; Cancer-Testis (CT) antigen), *CD44*, *IRF4* (Interferon Regulatory Factor 4, also known as Multiple Myeloma Oncogene 1 *[MUM1]*), *MFGE8/lactadherin*, *BCL2* (B-cell lymphoma 2), *CD146/MCAM* (Melanoma Cell Adhesion Molecule)/*MUC18*; *IGF1R* (insulin-like growth factor 1 receptor), *PNMA1* (paraneoplastic Ma antigen protein family; CT antigen); and *LGALS3/Galectin-3* (Figure 3A, Table S3A, and Figure S2A).

Furthermore, DE analysis comparing nBAP1-ve versus nBAP1+ve mUM revealed significant downregulation of *AKT3* (adjusted *p* < 0.05) (Figure 3B and Table S3B). A number of other antigen processing–related, cytokine, chemokine, T-cell and NK-cell function related genes were downregulated in nBAP1-ve mUM but did not reach statistical significance (Figure 3B and Table S3B).

**Figure 4:**
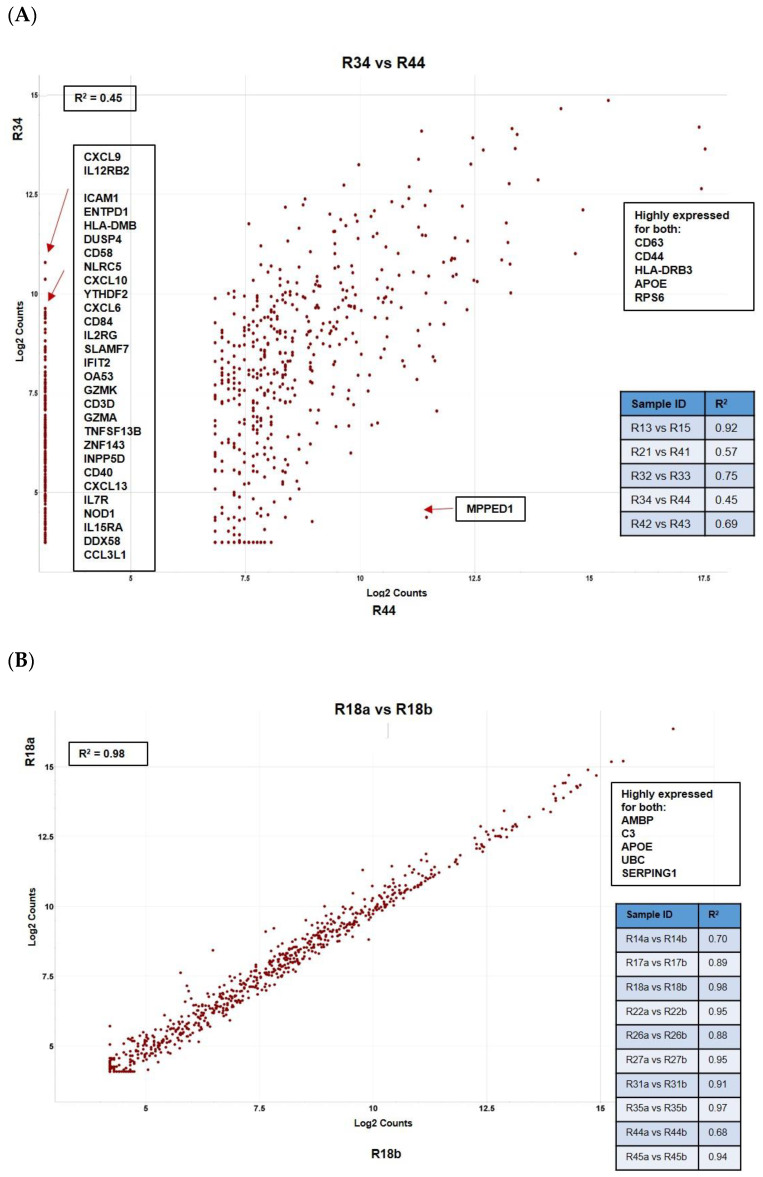
Pairwise ratio comparisons of normalized data demonstrating: (**A**) intersegmental heterogeneity for hepatic mUM taken from the same patient where marked difference in a number of immune-related genes was noted and (**B**) between two histomorphologically distinct areas within the same tumour deposit (intratumoural analysis) showing lack of transcriptomic variability. All results are presented as scattered plots with the “R^2^” coefficients given for each of the comparisons. Genes consistently highly expressed between samples are highlighted.

The pairwise comparisons of *Intersegmental mUM from the same patient* (representative scatter plot in Figure 4A) showed that there was significant variability of all immune-related genes between the different mUM lesions for each case. The genes consistently highly expressed in mUM from different liver segments included: *HLA-A*, *HLA-B*, *HLA-C* (major histocompatibility class I), and HLA-DRB3 (major histocompatibility class II); *CD63* and *CD44*; *APOE*; *RPS6* (ribosomal protein S6); and *AMBP* (Alpha-1-Microglobulin). This contrasted the pairwise ratio analyses of the *intratumoural mUM*, comparing microdissected areas of the same mUM lesion from the same patient—the representative scatter plot in Figure 4B shows a high correlation between the NanoString expression profiles.

### 2.3. Immunohistochemical Studies

**Figure 5:**
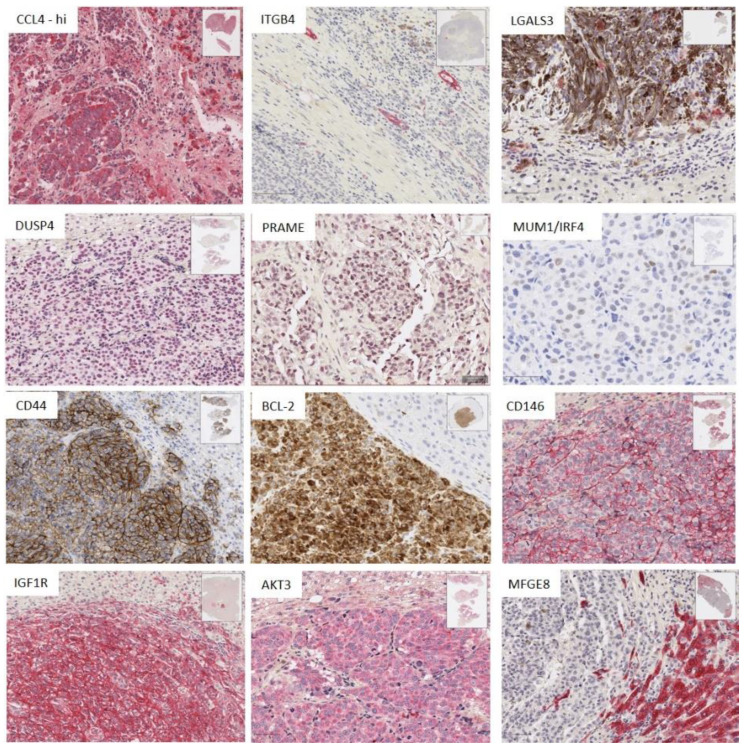
Immunohistochemistry (IHC) results showing the staining pattern and spatial distribution of positive cells for antibodies against the proteins encoded by genes of interest identified from NanoString analyses (scale bar: 100 µm for CCL4, ITGNB4, and DUSP4. All other IHC panels scale bar: 50 µm). (Table S4 summarizes the key findings).

IHC stains were undertaken where possible for: (a) the inflammatory markers of note that were *upregulated* by at least a log_2_ fold change of 2 in the DE analysis between mUM of differing immunoscores (i.e., CCL4) or substantially *downregulated* (i.e., ITGB4) and (b) for the genes highlighted in the DE analysis of mUM versus hepatocytes, as well as in the intersegmental and intratumoural analyses (i.e., DUSP4, PRAME, CD44, MUM1/IRF4, MFGE8, BCL-2, CD146/MCAM/MUC1, IGF1R, PNMA, and LGALS3). The results are seen in Figure 5 and summarized in Table S4.

CCL4 was highly expressed (75–100% of positive staining cells) by both tumour cells and TAMs in TAM-high mUM. In contrast, ITGB4 demonstrated positivity only in bile ducts and ductules (Figure 5 and Table S4). Its downregulation in the mUM samples was associated with the destruction of these structures within or at the edge of the mUM and associated inflammation or necrosis.

Marked overexpression of DUSP4, CD44, MUM1/IRF4, BCL-2, CD146/MUC18, IGF1R, and AKT3 was observed in the tumour cells in all mUM, compared to the background hepatocytes (Figure 5 and Table S4). PRAME expression was observed in 75–100% of the tumour cells of two mUM only. There was no difference in the expression of the above proteins between nBAP+ve or nBAP1-ve mUM.

**Figure 6:**
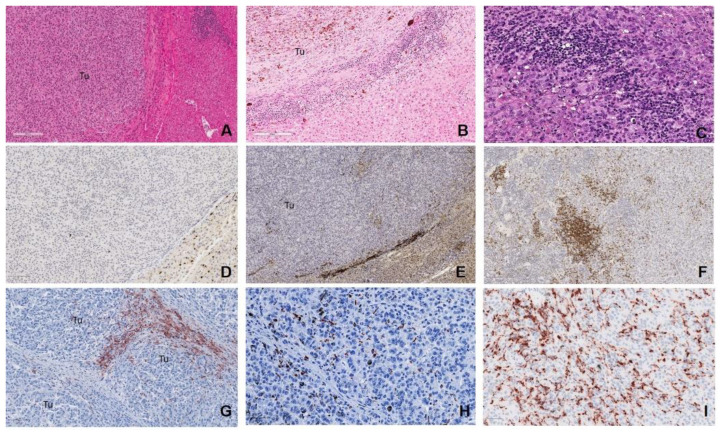
Grading of tumour immunoscore. H&E photomicrographs demonstrating: (**A**) “absent/cold” tumour with no TILs or TAMs within the tumour (Tu) or at the tumour-normal liver interface, given a “low” immunoscore (scale bar: 200 µm). The “intermediate/altered” categories: (**B**) “altered excluded,” where TILs or TAMs infiltrates were low at the tumour centre (Tu) and high at the margin (scale bar: 200 µm); “altered immunosuppressive,” a low scattered pattern of TILS/TAMS (not shown here). (**C**) “High/hot,” with marked TILs or TAMs infiltration throughout the metastatic deposit (scale bar: 50 µm). (**D**–**F**) CD8+ TILs for each of the categories, respectively (scale bar: 100 and 200 µm, respectively). CD163+ TAMs for the categories: (**G**) “altered excluded,” (scale bar: 100 µm) (**H**) “altered immunosuppressed” and (**I**) “high” (scale bar: 50 µm).

LGALS3 protein expression was consistently seen in the macrophages within the mUM (Figure 6 and Table S4). Nuclear IRF4/MUM1 expression was also seen in some mUM with scattered plasma cells (not shown), typically in areas of desmoplasia and necrosis. MFGE8 was not expressed by the melanoma cells, rather it was expressed variably by the hepatocytes, with those directly adjacent to the mUM showing strong expression of this protein. Interestingly, the bile ducts within the metastases and the normal parenchyma were also strongly positive for MFGE8 with some mUM showing a proliferation of bile ducts in the tumour within areas of desmoplasia (Figure 5 and Table S4). Despite repeated attempts, PNMA protein expression could not be demonstrated in FFPE using any of the commercially available antibodies.

## 3. Discussion

Our study is the first to undertake NanoString transcriptome profiling on a large cohort of hepatic mUM, which were clinically and morphologically phenotyped and also defined by applying a modified immunoscore classifier, with the results subsequently being validated using IHC. Further, we interrogated our mUM cohort for inter- and intratumoural transcriptome heterogeneity, because of its potential therapeutic relevance. Our data reveal new biomarkers expressed by mUM and their TME, which are worthy of further pursuit in the search for targeted therapies in this fatal disease.

With respect to the immune profile of the mUM, we confirm our previous results [25,26] that the main “reactive inflammatory” cell within these metastatic tumours is the M2 macrophage with an expression of CD68, CD163, and CD4. Although considered to be bystander cells, these TAMs are likely to actively contribute to the immunosuppressive environment within the mUM. Our current study demonstrated that the M2 TAMs were characterized by an upregulation of *ANXA1*, *CD74*, *CXCR4*, *MIF*, *STAT3*, *PLA2G6*, and *TGFB1* (Figure S1), all of which are involved in anti-inflammatory activity. We also noted high expression of *LGALS3* (Figure 4A) and *HLA-DRA* (not shown) and gene markers for immunosuppression (Figure 2B), as has been previously shown in primary UM [26].

Interestingly, those mUM with a high TAM immunoscore expressed high levels of several chemokines, namely, *CCL4* (MIP-1β), *CCL3* (MIP-1α), and *CCL3L1* (involved in the CCR5 macrophage pathway), some of which are released by activated hepatic stellate cells (HSC) [32], suggesting that there may be crosstalk between the two cell populations, ultimately promoting tumour angiogenesis and fibrosis. The importance of a bidirectional crosstalk between mUM cells and HSC in promoting mUM colonization and preventing inflammatory cell infiltration has been previously described by ourselves and others [33,34,35,36] and may prove useful as a strategy in the development of mUM therapies.

Other “players” in the immune profile of the mUM were DCs followed by CD8+ T-lymphocytes, B-cells, plasma cells, and NK cells. DCs have been previously described in mUM [27,37,38]; indeed, it was hoped that as antigen-presenting cells, they could activate antigen-specific T-cells directed against mUM and, therefore, result in anti-tumour immune activity. This formed the basis of some DC vaccination trials, which showed a response in ~30% of patients [39]. However, UM cells can inhibit DC immunostimulatory function, and it would appear that DCs in the context of high tumour volume mUM are incapable of stimulating an effective immune response [40]. Hence, trials were established to treat high-risk UM patients (identified by monosomy 3 of the primary tumour) with DC vaccinations in an adjuvant setting after resection of the primary tumour [41,42]. A slight improvement in overall survival rates was achieved in patients with a detectable tumour antigen-specific immune response after DC vaccination.

As previously described [25,27,28,43], the predominant lymphocyte population present within most mUM were exhausted CD8+ TILs (82.5%) showing either an “altered excluded” or “altered immunosuppressed” pattern, corresponding to a low or intermediate immunoscore, respectively. Interestingly, those mUM with a “high” TIL immunoscore had upregulation of the *CD25/ILR2A*, corresponding to regulatory T-cells (Tregs), and thereby creating a TME characterized by immunosuppression directed against tumour antigen-specific effector T cells, B cells, and plasma cells, ultimately leading to immune exhaustion and/or anergy [44,45,46,47]. Indeed, in this study, very few mUMs contained B-cells and plasma cells. The latter cell type was seen particularly in those mUM that had undergone necrosis and were associated with a desmoplastic reaction (Figure 6). Plasma and CD20+ B-cells have been described in primary UM, albeit as focal aggregates in small quantities, and in some cases associated with necrosis [26,48,49,50]. Our observations may thus explain the relatively poor responses to trialled adoptive transfer of autologous TIL-based therapies in patients with mUM [51] and emphasise the urgent need to refine T-cell therapy in this setting [52].

Corroborating previous literature, our analyses also confirm low expression of many ICRs in mUM (e.g., CTLA4 and PD1) [24,26,52] and provide an explanation as to why current immunotherapies are ineffective in mUM [22,52]. Interestingly, however, we did note upregulation of *CD276/B7-H3*, *HMGB1*, and CD73/*NT5E* in mUM, but these were also seen in the control liver, making them inappropriate to pursue as therapeutic targets in hepatic mUM (Supplementary Figure S5B). This is in contrast to other human malignancies, including cutaneous melanoma, where these 3 alternate ICRs are preferentially expressed on tumour cells and are currently in phase I clinical trials [53,54,55]. Our results importantly highlight yet again the differences between mUM and metastatic skin melanoma and the urgent need to look for alternate targets and receptors in mUM [44,45,46,47], which may be specifically found on the tumour cells rather than within the TME, as discussed below.

Compared to background liver, mUM significantly increased expression of 11 genes (adjusted *p* < 0.01), of which 10 had a log_2_ fold-change greater than 2 in our transcriptomic analyses. Some of these genes have not been previously described, and they may serve as new directions for targeted therapy in mUM.

The most highly expressed gene in mUM was *DUSP4*, also known as mitogen-activated protein kinase phosphatases 2 (MKP2), located on chromosome 8p12-p11 [56]. It belongs to the DUSP family, which is responsible for dephosphorylation and inactivation of mitogen-activated protein kinase (MAPK) family members [57,58]. DUSPs exhibit substrate specificity towards particular MAPKs, with *DUSP4* regulating ERK1, ERK2, p38, and c-Jun N-terminal kinase [59], which makes it potentially associated with three of the four main MAPK signalling pathways [60]. Although *DUSP4* expression has been reported previously in skin melanomas, and as a potential biomarker for patient response to MEK inhibition with its upregulation correlating with a positive response [61,62], it has not been described previously in UM. Analysis of the publicly available TCGA dataset reveals that DUSP4 mRNA was overexpressed in 4% of the cases (3 out of 80 cases) [63,64,65]. The fact that DUSP4 may not commonly be overexpressed in primary UM may suggest that its enhanced expression is specific for mUM, as in other cancers [66]; however, this requires further investigation. Although there are no trials targeting DUSP4, siRNA depletion of DUSP4 sensitizes cancer cell lines to drugs to which they were otherwise resistant [67,68], a strategy that may be of value in mUM.

Another novel finding of our study was the overexpression of *IRF4/MUM1* in mUM. The *IRF4* gene (chr 6p25.3) belongs to a family of transcription factors for interferons; therefore, they are involved in the regulation of the immune system and oncogenesis [69]. IRF4 is involved in the differentiation and maturation of B-cells, plasma cells, as well as memory T-cells, and hence, most clinical trials targeting this molecule are associated with lymphomas or plasma cell neoplasms. Interestingly, and perhaps relevant for the M2-rich mUM, IRF4 together with IRF8 promotes differentiation of the myeloid progenitor cells to macrophages [70]. IRF4 is also associated with the macrophage polarization process: together with upstream JMJD3 (Jumonji domain-containing protein D3), it is responsible for controlling M2 macrophage marker expression [71]. Although IRF4 expression has been described in skin melanoma [72,73,74], it has not been previously reported in either primary or metastatic UM. Our interrogation of TCGA data set (Firehose Legacy) reveals that *IRF4* is not only amplified in 10% of the samples but also overexpressed at the mRNA level in 14% of the samples [63,64,65]. Considering that the *IRF4* gene is localized on chromosome 6p, which is usually associated with a good prognosis, correlation of IRF4/MUM1 protein expression in primary UM and the tumour cell copy number variations may be of value for future use in diagnostic laboratories.

Paraneoplastic Ma1 (*PNMA1*) is a member of an expanding family of “brain/testis” proteins thought to be involved in an autoimmune disorder defined as paraneoplastic neurological syndrome. However, the biological and clinical significance of PNMA1 in tumours is poorly understood. Although increased *PNMA1* expression and its protein levels have been shown to be proapoptotic in neurons [75], they have been found to play a prosurvival and antiapoptotic role in pancreatic [76], gastric [77], and breast cancers [78]. Till date, there is no literature on the role of PNMA1 in melanoma or UM. Although clearly upregulated in mUM at the transcriptome level, disappointingly, we were not able to validate this at the protein level using the currently available antibodies using IHC.

Our data also corroborated results of previous studies. For example, mUM demonstrated significant overexpression of *PRAME* at the mRNA level. *PRAME* expression has been previously described in primary and mUM [79,80,81]. Interestingly, however, PRAME expression using IHC was not strong with only 2/19 samples demonstrating focal immunoreactivity. *PRAME* (chr. 22q11.22) is a CT antigen not expressed on normal tissues and is a transcriptional repressor of the retinoic acid receptor; it thereby inhibits retinoic acid induced proliferation arrest and apoptosis, which gives cancer cells survival and growth advantages [82]. Given its preferential expression in cancer cells, PRAME attracted a lot of interest as a potential immunotherapy target. Two main therapeutic approaches in PRAME-targeted therapy include cancer vaccines and adoptive T cell therapy. The status of finished and ongoing clinical trials targeting PRAME has been recently reviewed [83]. This includes a phase 1/2 dose-finding and -expansion trial for mUM patients that commenced in 2017 whereby participants’ T-cells are modified to recognize and target PRAME (https://clinicaltrials.gov/ct2/show/NCT02743611?term=PRAME&draw=2&rank=1).

*CD44* (chr 11p13) demonstrated similar levels of mRNA overexpression as *PRAME* in the mUM and is involved in a wide variety of cellular functions. It encodes a cell-surface glycoprotein involved in cell–cell interactions, cell adhesions, and migration. CD44′s main ligand is hyaluronic acid, which is an abundant component of extracellular matrix, and its binding results in induced cell proliferation, increased cell survival, and enhanced cell motility [84]. High expression of CD44, including some of its alternatively spliced variants, is seen in cancer stem cells and is thought to play a role in cancer development and progression [85]. CD44 expression has been noted in three UM cell lines and primary UM [86,87], however, to date has not been reported in mUM. The distinctive and strong expression of CD44 in mUM cells could place it as a candidate for the detection of circulating mUM cells in patient sera. CD44, particularly its hyaluronan receptor [88], has attracted a lot of interest as a potential therapeutic target for many solid tumours; however, no trials at present are being undertaken in mUM.

Distinctive and strong expression of *CD146/MCAM/MUC18* was noted at both the mRNA and protein level in our cohort of mUM. Localized on chromosome 11q23.3, *CD146* codes for a cell adhesion protein, which belongs to the immunoglobin family, with its main role being the facilitation of cell adhesion through binding with neighbouring cells or with the extracellular matrix [89]. Our data support those of previous groups, who have demonstrated strong CD146 expression on primary and mUM [90,91], as well as on circulating UM cells [92,93,94]. Interestingly, CD146 is the major galectin-3-binding ligand and colocalizes with galectin-3 on endothelial cell surfaces, inducing AKT activation and thereby leading to cell survival, growth, proliferation, cell migration, and angiogenesis, as well as the secretion of metastasis-promoting cytokines [95]. Our preliminary studies on UM cell lines also show that galectin 3 colocalizes with CD146 on their cell membranes, and that introduction of exogenous galectin-3 to these cultured cells induces a dose-dependent increase of AKT phosphorylation (Prof. Lugang Yu, University of Liverpool, unpublished results). Recent developments in the production of galectin 3 inhibitors [96] could, therefore, hold promise for the treatment of mUM.

The *BCL2* gene (chr. 18q21.33) encodes for BCL-2, the first protein to be described in the BCL-2 family, which regulate cell death by either inhibiting (antiapoptotic) or inducing (proapoptotic) apoptosis [97]. BCL-2 is antiapoptotic and its overexpression has been described in most primary UM [98,99,100,101,102]. However, no particular correlation with histomorphological or genetic features of the tumours nor with clinical outcome have been reported. Although not previously investigated in such a large patient mUM cohort until the current study, BCL2 has been the subject of studies to improve the cytotoxic effect of different chemotherapeutics against UM cells in preclinical settings [103,104,105,106]. Indeed, Decaudin et al. performed a comprehensive preclinical screening of a panel of 5 different BCL2 protein family inhibitors and assessed their synergy with PI3K/AKT/mTOR, p53, and MAPK/ERK inhibitors against both primary and metastatic UM cell lines. Several combinations showed a synergetic effect, and the most promising combinations were further assessed in patient-derived xenograft (PDX) models. The BCL-2/XL/W inhibitor (ABT263) combined with MDM2 inhibitor (HDM201) showed a trend for a synergistic effect in the PDX models [106]. However, further studies employing BCL-2 in the context of mUM are required considering data that suggest high galectin-3 expression, as seen in mUM, may mediate resistance to BCL-2 targeted therapies [107].

*IGF1R* was strongly and distinctively overexpressed at both the transcriptome and protein levels in mUM in our study. This confirms previous observations in primary UM cell lines and UM samples [108,109,110,111] as well as in mUM [112]. Insulin-like growth factor-1 is a strong mitogen, which on stimulating IGF-1R signalling plays an important role in UM development and spread [113]. Hence, targeting IGF signalling has been considered a promising approach to inhibit the process of metastatic UM cells, e.g., recent studies have looked at this pathway using single [113] and a combinatory targeted approach [114].

With respect to tumour heterogeneity with mUM, there was little variation in the transcriptomic expression profiles for immune-related and tumour-cell-related genes within a single UM metastasis. In contrast, however, there were significant transcriptome differences between mUM lesions within the same patient with respect to immune-related genes. These data correlated with the respective morphological findings where inflammatory infiltrates within and surrounding mUM were dependent on the presence/absence of necrosis, haemorrhage, and desmoplasia. Our data (particularly those of mUMs R4, R34, and R35 which arose from the same patient) support those of previous researchers suggesting that there may be differing clones within unusual cases of UM and that there could be evolution of genetic alterations in mUM with time [29,115]. These findings suggest that heterogeneity of the TME associated to mUM lesions could affect the response to therapies.

## 4. Materials and Methods

The study was approved by the Health Research Authority (REC Ref 11/NW/0759) and conducted in accordance with the Declaration of Helsinki. All samples were provided by the Ocular Oncology Biobank (REC Ref 16/NW/0380) and the Liverpool Bio-Innovation Hub Biobank following local approval.

### 4.1. Specimen

Archival formalin-fixed paraffin embedded (FFPE) mUM specimens (n = 40) were obtained from 27 consented patients who had undergone resection of their liver metastases at Aintree University Hospital, Liverpool between 2003 and 2017. All samples were assessed by Y.K., A.A.S., H.K., and S.E.C., and the hepatic metastases classified for the following: dominant cell type growth pattern [43], cell morphology, degree of pigmentation, presence/absence of necrosis, and nuclear BAP1 (nBAP1) immunoreactivity. Information on chromosome 3 status of the primary UM [3,10,116,117] for each patient was taken from previous data where available. Noted also were the presence/absence of fibrous encapsulation of mUM, the degree of intratumoural fibrosis within the metastatic deposit, as well as the presence/absence of intertumoural “bridging” fibrosis (Figure S4). The degree of inflammation associated with each mUM sample was defined by an “immunoscore,” as previously described by Galon et al. [118,119,120] (Figure 6), and is outlined in detail under “Immunohistochemistry” below. The normal tissue blocks associated with the liver resections of six UM patients, confirmed to be devoid of tumour cells by both H&E and immunohistochemistry (IHC), were pooled and included as controls (CL1 and CL2: each containing 3 control livers).

### 4.2. RNA Isolation, Quantification, and Quality Assessment

Four fresh 5 µm sections cut from the FFPE hepatic mUM were deparaffinized using xylene, homogenized through vortexing, and incubated at 56 °C for 2 h and then 80 °C for 15 min on a ThermoMixer^®^ C (Eppendorf UK Ltd., Stevenage, UK). Total RNA was prepared using the RNeasy FFPE Kit (QIAGEN Ltd., Manchester, UK) as per the manufacturer’s protocol. For the 0.6 mm core punches taken for the third experimental arm (see below), a further homogenization step was undertaken during deparaffinization using tissue grinder pestles. Total RNA purity and quantity were assessed by Nanodrop spectrophotometry (Thermo Fisher Scientific, Life Technologies Ltd. Paisley, UK) and with the Qubit^®^ RNA Broad-Range Assay Kit and fluorometer (Molecular Probes, Life Technologies Ltd. Paisley, UK), respectively.

### 4.3. Transcriptome Analyses

Gene expression profiling was performed using the nCounter^®^ PanCancer Immune Profiling Panel Kit (NanoString Technologies, Seattle, WA, USA). The transcriptomic analyses were divided into three separate components:

(1) *mUM versus control liver*—all 40 mUM specimens (R1 to R40; Table S1) with 6 control liver samples (which had been pooled to create 2 representative controls (CL1 and CL2: each containing 3 control livers) were used across all cartridges;
(2) *Intersegmental mUM analysis*—comparison of mUM resections from two different liver segments of the same patient (n = 5 patients, giving a total of 10 different mUM samples (Table S1));
(3) *Intratumour mUM analysis*—two different areas within the same hepatic mUM nodule (n = 10 mUM resections (Table S1)) were taken using 0.6 mm core punches (2 mm × 0.6 mm diameter from each region of interest; n = 20 regions of interest) (Figure S5).

In each study component, 250 ng total RNA for each specimen was hybridized with the Immune Profiling nCounter^®^ CodeSet (reporter and capture probes) as per the manufacturer’s instructions. The CodeSet included 730 immune-related genes (immune cell types markers, checkpoint inhibitors, cancer-testis antigens, and genes involved in adaptive and innate immune responses) and 40 house-keeping genes. The expression of each of these 770 genes was interrogated within each of the samples. The CodeSet also included six positive and six negative controls. After hybridization, samples were loaded onto the nCounter^®^ FLEX System prep station (NanoString Technologies) for cartridge processing, and cartridges were then scanned by the multichannel epifluorescence nCounter^®^ Digital Analyser (NanoString Technologies) following the manufacturer’s recommendations. The latter utilizes digital detection of direct fluorescent molecular barcodes.

### 4.4. Data Analysis and Bioinformatics

Comprehensive transcriptomic data mining was performed using the nSolver™ 4.0 data analysis software with nCounter Advanced Analysis Module 2.0 (NanoString Technologies). Briefly, quality control (QC; binding density, image quality, assay efficiency, assay linearity, and limit of detection) was done for each sample, and any samples raising a QC flag were excluded. For mUM versus control liver, QC flags were raised in three of the mUM and these RNA samples were excluded from the analyses: R15 and R31 (normalization flags) and R38 (binding density flag). There were no QC flags raised in the intra- and intertumoural study components.

For mUM versus control, data were normalized using the widely accepted in-built geNorm algorithm. Sample R18 was found to be an outlier after geNorm normalization and was excluded (R18 was profiled independently and was not an outlier in the other study components). All statistical analyses were completed using R scripts available within the nCounter Advanced Analysis Module. Multivariate regression analyses were performed within the Advanced Analysis Module with Benjamini-Hochberg *p*-value adjustment, and results were presented as differential expression volcano plots showing log_2_ fold changes in gene expression in the mUM versus control liver. Genes of interest (Figure S3) were also examined using heatmaps (presented individually and also as the sum of normalized expression for each functional category).

For intersegmental and intratumour mUM comparisons, two-step normalization using the positive control and house-keeping genes followed by background thresholding against the geometric mean of negative controls was undertaken prior to analysis with the nSolver software. Normalized data were represented as a series of scatter plots on which genes of interest and “R^2^” coefficients were indicated.

### 4.5. Immunohistochemical Validation of NanoString Transcriptome Analyses

Sections were cut at 4 µm from the FFPE blocks of hepatic mUM used in the NanoString analyses onto X-tra™ adhesive slides (Leica) where sufficient tissue was remaining (n = 19) and processed for IHC as previously described [25,29]. Briefly, antigen retrieval and IHC were performed using the Bond RXm Automated Stainer with the Bond polymer refine detection systems in either red or brown, according to the manufacturers’ recommendations (Leica Biosystems UK Ltd., Milton Keynes, UK). Primary antibodies (Abs) against the genes of interest (statistically significant log_2_ fold-changes at least >2) revealed through the NanoString analysis—i.e., *DUSP4*, *PRAME*, *CD44*, *IRF4/MUM1*, *BCL2*, *CD146/MCAM/MUC18*, *IGF1R*, *PNMA1*, *MFGE8*, *LGAL3/Galectin-3*, *ITGB4*, *CD25/IL2RA*, and *AKT3*—were examined using IHC (Table S5). Positive and negative controls for each of the primary antibodies were included in each assay. Slides were mounted with a resin-based mountant. Previous IHC performed as part of the routine clinical work-up for mUM was also re-evaluated and included: MelanA, BAP1, CD3, CD4, CD8, CD68, CD163, and CD20 (as previously described) [25,121].

### 4.6. Grading of Inflammation

All mUM samples were assessed for the density, spatial and cellular distribution of CD3+/CD8+ TILs and CD68+/CD163+ TAMs, and an immunoscore grading given, as previously described by Galon et al. [118,119,120]. Briefly, infiltration of TILs and TAMs differentiated the mUM TME into four distinct inflammatory response phenotypes: “absent/cold,” where the metastasis was devoid of these cells; “altered excluded,” where TILs or TAMs infiltrates were low at the tumour centre and high at the peritumoural margin; “altered immunosuppressive,” where mUM displayed a low scattered pattern of inflammatory cell infiltrate; or “high/hot,” with marked TILs or TAMs infiltration throughout the metastatic deposit (Figure 6). These inflammatory response phenotypes were given an immunoscore of “low (absent) (0),” “intermediate (altered category) (1),” or “high (2)”, respectively [120], for both TILS and TAMS. A total immunoscore for each mUM was generated by summing the two values; the score range of “0–3” was denoted a “low” total immunoscore, and the range of “4–6” was indicated a “high” total immunoscore.

## 5. Conclusions

In summary, we have confirmed the M2 macrophage being the dominant cell type within mUM with most of these metastases having low or intermediate immunoscores for T-cells. The TAMs are characterized by the upregulation of a transcriptomic signature, which enhance an immunosuppressed environment. We have also demonstrated the expression of novel markers in mUM, some of which are specifically and highly expressed in the tumour cells only and thereby, could potentially represent possible targets for single and/or combinatory therapy.

## Supplementary Materials

The following are available online at https://www.mdpi.com/2072-6694/12/10/2832/s1, Figure S1: Heatmap highlighting the relative expression of gene markers for M2 TAMs in mUM versus control; Figure S2: Heatmaps showing relative gene expression within mUM versus control for: (A) cancer-testis antigens and (B) immune checkpoint inhibitors; Figure S3: Venn diagrams showing the list of genes used for immune cell type profiling (A) and cell function profiling (B); Figure S4: Increasing stages of fibrosis, and necrosis; Figure S5: Macroslide image of a hepatic mUM demonstrating how cores were taken for intratumoural heterogeneity analysis. Table S1: Clinical and histomorphological information for all the hepatic mUM samples; Table S2: NanoString differential expression output data for mUM for: (A) Total immunoscore, (B) TAMs immunoscore, (C) TILs “altered versus absent,” and (D) TILs “high versus absent;” Table S3: NanoString differential expression output data for mUM for: (A) all mUM versus control liver and (B) nBAP1 status in mUM; Table S4: Summary of IHC results; Table S5: Details of the antibodies used for IHC.

## References

[1] Kujala E., Makitie T., Kivela T. (2003). Very long-term prognosis of patients with malignant uveal melanoma. Investig. Ophthalmol. Vis. Sci..

[2] Damato B. (2012). Progress in the management of patients with uveal melanoma. The 2012 Ashton Lecture. Eye (Lond).

[3] Damato B., Dopierala J., Klaasen A., van Dijk M., Sibbring J., Coupland S.E. (2009). Multiplex ligation-dependent probe amplification of uveal melanoma: Correlation with metastatic death. Investig. Ophthalmol. Vis. Sci..

[4] Kaliki S., Shields C.L., Shields J.A. (2015). Uveal melanoma: Estimating prognosis. Indian J. Ophthalmol..

[5] Sato T., Babazono A., Shields J.A., Shields C.L., De Potter P., Mastrangelo M.J. (1997). Time to systemic metastases in patients with posterior uveal melanoma. Cancer Investig..

[6] Robertson A.G., Shih J., Yau C., Gibb E.A., Oba J., Mungall K.L., Hess J.M., Uzunangelov V., Walter V., Danilova L. (2017). Integrative Analysis Identifies Four Molecular and Clinical Subsets in Uveal Melanoma. Cancer Cell.

[7] Royer-Bertrand B., Torsello M., Rimoldi D., El Zaoui I., Cisarova K., Pescini-Gobert R., Raynaud F., Zografos L., Schalenbourg A., Speiser D. (2016). Comprehensive Genetic Landscape of Uveal Melanoma by Whole-Genome Sequencing. Am. J. Hum. Genet..

[8] Thornton S., Coupland S.E., Olohan L., Sibbring J.S., Kenny J.G., Hertz-Fowler C., Liu X., Haldenby S., Heimann H., Hussain R. (2020). Targeted Next-Generation Sequencing of 117 Routine Clinical Samples Provides Further Insights into the Molecular Landscape of Uveal Melanoma. Cancers.

[9] Coupland S.E., Damato B.E. (2013). Molecular analysis of uveal melanoma. Ophthalmology.

[10] Coupland S.E., Lake S.L., Zeschnigk M., Damato B.E. (2013). Molecular pathology of uveal melanoma. Eye (Lond).

[11] Coupland S.E., Thornton S., Kalirai H. (2020). Importance of Partial Losses of Chromosome 3 in Uveal Melanoma in the BAP1 Gene Region. JAMA Ophthalmol..

[12] Damato B., Coupland S.E. (2009). Translating uveal melanoma cytogenetics into clinical care. Arch. Ophthalmol..

[13] Damato B., Coupland S.E. (2009). Genomic typing of uveal melanoma. Arch. Ophthalmol..

[14] Damato B., Duke C., Coupland S.E., Hiscott P., Smith P.A., Campbell I., Douglas A., Howard P. (2007). Cytogenetics of uveal melanoma: A 7-year clinical experience. Ophthalmology.

[15] Harbour J.W., Onken M.D., Roberson E.D., Duan S., Cao L., Worley L.A., Council M.L., Matatall K.A., Helms C., Bowcock A.M. (2010). Frequent mutation of BAP1 in metastasizing uveal melanomas. Science.

[16] Kalirai H., Dodson A., Faqir S., Damato B.E., Coupland S.E. (2014). Lack of BAP1 protein expression in uveal melanoma is associated with increased metastatic risk and has utility in routine prognostic testing. Br. J. Cancer.

[17] Damato B. (2001). Time to treatment of uveal melanoma in the United Kingdom. Eye (Lond).

[18] Furney S.J., Pedersen M., Gentien D., Dumont A.G., Rapinat A., Desjardins L., Turajlic S., Piperno-Neumann S., de la Grange P., Roman-Roman S. (2013). SF3B1 mutations are associated with alternative splicing in uveal melanoma. Cancer Discov..

[19] Martin M., Masshofer L., Temming P., Rahmann S., Metz C., Bornfeld N., van de Nes J., Klein-Hitpass L., Hinnebusch A.G., Horsthemke B. (2013). Exome sequencing identifies recurrent somatic mutations in EIF1AX and SF3B1 in uveal melanoma with disomy 3. Nat. Genet..

[20] Gomez D., Wetherill C., Cheong J., Jones L., Marshall E., Damato B., Coupland S.E., Ghaneh P., Poston G.J., Malik H.Z. (2014). The Liverpool uveal melanoma liver metastases pathway: Outcome following liver resection. J. Surg. Oncol..

[21] Sato T. (2010). Locoregional management of hepatic metastasis from primary uveal melanoma. Semin. Oncol..

[22] Sacco J.J., Kalirai H., Kenyani J., Figueiredo C.R., Coulson J.M., Coupland S.E. (2018). Recent breakthroughs in metastatic uveal melanoma: A cause for optimism?. Future Oncol..

[23] Robert C., Schachter J., Long G.V., Arance A., Grob J.J., Mortier L., Daud A., Carlino M.S., McNeil C., Lotem M. (2015). Pembrolizumab versus Ipilimumab in Advanced Melanoma. N. Engl. J. Med..

[24] Komatsubara K.M., Carvajal R.D. (2017). Immunotherapy for the Treatment of Uveal Melanoma: Current Status and Emerging Therapies. Curr. Oncol. Rep..

[25] Krishna Y., McCarthy C., Kalirai H., Coupland S.E. (2017). Inflammatory cell infiltrates in advanced metastatic uveal melanoma. Hum. Pathol..

[26] Figueiredo C.R., Kalirai H., Sacco J.J., Azevedo R.A., Duckworth A., Slupsky J.R., Coulson J.M., Coupland S.E. (2020). Loss of BAP1 expression is associated with an immunosuppressive microenvironment in uveal melanoma, with implications for immunotherapy development. J. Pathol..

[27] Borthwick N.J., Thombs J., Polak M., Gabriel F.G., Hungerford J.L., Damato B., Rennie I.G., Jager M.J., Cree I.A. (2011). The biology of micrometastases from uveal melanoma. J. Clin. Pathol..

[28] Barnhill R., Vermeulen P., Daelemans S., van Dam P.J., Roman-Roman S., Servois V., Hurbain I., Gardrat S., Raposa G., Nicolas A. (2018). Replacement and desmoplastic histopathological growth patterns: A pilot study of prediction of outcome in patients with uveal melanoma liver metastases. J. Pathol. Clin. Res..

[29] McCarthy C., Kalirai H., Lake S.L., Dodson A., Damato B.E., Coupland S.E. (2016). Insights into genetic alterations of liver metastases from uveal melanoma. Pigment Cell Melanoma Res..

[30] Spencer J., MacDonald T.T., Finn T., Isaacson P.G. (1986). The development of gut associated lymphoid tissue in the terminal ileum of fetal human intestine. Clin. Exp. Immunol..

[31] Waks A.G., Stover D.G., Guerriero J.L., Dillon D., Barry W.T., Gjini E., Hartl C., Lo W., Savoie J., Brock J. (2019). The Immune Microenvironment in Hormone Receptor-Positive Breast Cancer Before and After Preoperative Chemotherapy. Clin. Cancer Res..

[32] Van den Eynden G.G., Majeed A.W., Illemann M., Vermeulen P.B., Bird N.C., Hoyer-Hansen G., Eefsen R.L., Reynolds A.R., Brodt P. (2013). The multifaceted role of the microenvironment in liver metastasis: Biology and clinical implications. Cancer Res..

[33] Angi M., Kalirai H., Prendergast S., Simpson D., Hammond D.E., Madigan M.C., Beynon R.J., Coupland S.E. (2016). In-depth proteomic profiling of the uveal melanoma secretome. Oncotarget.

[34] Babchia N., Landreville S., Clement B., Coulouarn C., Mouriaux F. (2019). The bidirectional crosstalk between metastatic uveal melanoma cells and hepatic stellate cells engenders an inflammatory microenvironment. Exp. Eye Res..

[35] Piquet L., Dewit L., Schoonjans N., Millet M., Berube J., Gerges P.R.A., Bordeleau F., Landreville S. (2019). Synergic Interactions Between Hepatic Stellate Cells and Uveal Melanoma in Metastatic Growth. Cancers.

[36] Coupland P.S., Ahmed I., Sakai T., Kalirai H. (2018). Liver fibrosis and metastatic uveal melanoma (mUM). Investig. Ophthalmol. Visual Sci..

[37] Ma D., Luyten G.P., Luider T.M., Niederkorn J.Y. (1995). Relationship between natural killer cell susceptibility and metastasis of human uveal melanoma cells in a murine model. Investig. Ophthalmol. Vis. Sci..

[38] Niederkorn J.Y. (1997). Immunoregulation of intraocular tumours. Eye (Lond).

[39] Bol K.F., Mensink H.W., Aarntzen E.H., Schreibelt G., Keunen J.E., Coulie P.G., de Klein A., Punt C.J., Paridaens D., Figdor C.G. (2014). Long overall survival after dendritic cell vaccination in metastatic uveal melanoma patients. Am. J. Ophthalmol..

[40] Ma J., Usui Y., Takeuchi M., Okunuki Y., Kezuka T., Zhang L., Mizota A., Goto H. (2010). Human uveal melanoma cells inhibit the immunostimulatory function of dendritic cells. Exp. Eye Res..

[41] Bol K.F., van den Bosch T., Schreibelt G., Mensink H.W., Keunen J.E., Kilic E., Japing W.J., Geul K.W., Westdorp H., Boudewijns S. (2016). Adjuvant Dendritic Cell Vaccination in High-Risk Uveal Melanoma. Ophthalmology.

[42] Schuler-Thurner B., Bartz-Schmidt K.U., Bornfeld N., Cursiefen C., Fuisting B., Grisanti S., Heindl L.M., Holbach L., Keseru M., Knorr H. (2015). Immunotherapy of uveal melanoma: Vaccination against cancer. Multicenter adjuvant phase 3 vaccination study using dendritic cells laden with tumor RNA for large newly diagnosed uveal melanoma. Ophthalmologe.

[43] Grossniklaus H.E., Zhang Q., You S., McCarthy C., Heegaard S., Coupland S.E. (2016). Metastatic ocular melanoma to the liver exhibits infiltrative and nodular growth patterns. Hum. Pathol..

[44] Grygorowicz M.A., Biernacka M., Bujko M., Nowak E., Rymkiewicz G., Paszkiewicz-Kozik E., Borycka I.S., Bystydzienski Z., Walewski J., Markowicz S. (2016). Human regulatory T cells suppress proliferation of B lymphoma cells. Leuk Lymphoma.

[45] Nishikawa H., Sakaguchi S. (2014). Regulatory T cells in cancer immunotherapy. Curr. Opin. Immunol..

[46] Tucci M., Passarelli A., Mannavola F., Felici C., Stucci L.S., Cives M., Silvestris F. (2019). Immune System Evasion as Hallmark of Melanoma Progression: The Role of Dendritic Cells. Front Oncol..

[47] Rothermel L.D., Sabesan A.C., Stephens D.J., Chandran S.S., Paria B.C., Srivastava A.K., Somerville R., Wunderlich J.R., Lee C.C., Xi L. (2016). Identification of an Immunogenic Subset of Metastatic Uveal Melanoma. Clin. Cancer. Res..

[48] Coupland S.E., Dodson A., Liu H., Du M.Q., Angi M., Damato B.E. (2013). Intraocular collision tumour: Case report and literature review. Graefes Arch. Clin. Exp. Ophthalmol..

[49] Durante M.A., Rodriguez D.A., Kurtenbach S., Kuznetsov J.N., Sanchez M.I., Decatur C.L., Snyder H., Feun L.G., Livingstone A.S., Harbour J.W. (2020). Single-cell analysis reveals new evolutionary complexity in uveal melanoma. Nat. Commun..

[50] Hussain R.N., Coupland S.E., Khzouz J., Kalirai H., Parsons J.L. (2020). Inhibition of ATM Increases the Radiosensitivity of Uveal Melanoma Cells to Photons and Protons. Cancers.

[51] Chandran S.S., Somerville R.P.T., Yang J.C., Sherry R.M., Klebanoff C.A., Goff S.L., Wunderlich J.R., Danforth D.N., Zlott D., Paria B.C. (2017). Treatment of metastatic uveal melanoma with adoptive transfer of tumour-infiltrating lymphocytes: A single-centre, two-stage, single-arm, phase 2 study. Lancet Oncol..

[52] Schank T.E., Hassel J.C. (2019). Immunotherapies for the Treatment of Uveal Melanoma-History and Future. Cancers.

[53] Picarda E., Ohaegbulam K.C., Zang X. (2016). Molecular Pathways: Targeting B7-H3 (CD276) for Human Cancer Immunotherapy. Clin. Cancer Res..

[54] Chen S., Wainwright D.A., Wu J.D., Wan Y., Matei D.E., Zhang Y., Zhang B. (2019). CD73: An emerging checkpoint for cancer immunotherapy. Immunotherapy.

[55] Guo Z.S., Liu Z., Bartlett D.L., Tang D., Lotze M.T. (2013). Life after death: Targeting high mobility group box 1 in emergent cancer therapies. Am. J. Cancer Res..

[56] Keyse S.M. (2000). Protein phosphatases and the regulation of mitogen-activated protein kinase signalling. Curr. Opin. Cell. Biol..

[57] Dickinson R.J., Keyse S.M. (2006). Diverse physiological functions for dual-specificity MAP kinase phosphatases. J. Cell. Sci..

[58] Theodosiou A., Ashworth A. (2002). MAP kinase phosphatases. Genome Biol..

[59] Keyse S.M. (2008). Dual-specificity MAP kinase phosphatases (MKPs) and cancer. Cancer Metastasis Rev..

[60] Morrison D.K. (2012). MAP kinase pathways. Cold Spring Harb. Perspect. Biol..

[61] Gupta A., Towers C., Willenbrock F., Brant R., Hodgson D.R., Sharpe A., Smith P., Cutts A., Schuh A., Asher R. (2020). Dual-specificity protein phosphatase DUSP4 regulates response to MEK inhibition in BRAF wild-type melanoma. Br. J. Cancer.

[62] Gupta R., Bugide S., Wang B., Green M.R., Johnson D.B., Wajapeyee N. (2019). Loss of BOP1 confers resistance to BRAF kinase inhibitors in melanoma by activating MAP kinase pathway. Proc. Natl. Acad. Sci. USA.

[63] Uveal Melanoma (TCGA, Firehose Legacy). https://www.cbioportal.org/study/summary?id=uvm_tcga.

[64] Cerami E., Gao J., Dogrusoz U., Gross B.E., Sumer S.O., Aksoy B.A., Jacobsen A., Byrne C.J., Heuer M.L., Larsson E. (2012). The cBio cancer genomics portal: An open platform for exploring multidimensional cancer genomics data. Cancer Discov..

[65] Gao J., Aksoy B.A., Dogrusoz U., Dresdner G., Gross B., Sumer S.O., Sun Y., Jacobsen A., Sinha R., Larsson E. (2013). Integrative analysis of complex cancer genomics and clinical profiles using the cBioPortal. Sci. Signal.

[66] Sim J., Yi K., Kim H., Ahn H., Chung Y., Rehman A., Jang S.M., Lee K.H., Jang K., Paik S.S. (2015). Immunohistochemical expression of dual-specificity protein phosphatase 4 in patients with colorectal adenocarcinoma. Gastroenterol. Res. Pract..

[67] Kang X., Li M., Zhu H., Lu X., Miao J., Du S., Xia X., Guan W. (2017). DUSP4 promotes doxorubicin resistance in gastric cancer through epithelial-mesenchymal transition. Oncotarget.

[68] Menyhart O., Budczies J., Munkacsy G., Esteva F.J., Szabo A., Miquel T.P., Gyorffy B. (2017). DUSP4 is associated with increased resistance against anti-HER2 therapy in breast cancer. Oncotarget.

[69] Nam S., Lim J.S. (2016). Essential role of interferon regulatory factor 4 (IRF4) in immune cell development. Arch. Pharm. Res..

[70] Yamamoto M., Kato T., Hotta C., Nishiyama A., Kurotaki D., Yoshinari M., Takami M., Ichino M., Nakazawa M., Matsuyama T. (2011). Shared and distinct functions of the transcription factors IRF4 and IRF8 in myeloid cell development. PLoS ONE.

[71] Satoh T., Takeuchi O., Vandenbon A., Yasuda K., Tanaka Y., Kumagai Y., Miyake T., Matsushita K., Okazaki T., Saitoh T. (2010). The Jmjd3-Irf4 axis regulates M2 macrophage polarization and host responses against helminth infection. Nat. Immunol..

[72] Natkunam Y., Warnke R.A., Montgomery K., Falini B., van De Rijn M. (2001). Analysis of MUM1/IRF4 protein expression using tissue microarrays and immunohistochemistry. Mod. Pathol..

[73] Ordonez N.G. (2014). Value of melanocytic-associated immunohistochemical markers in the diagnosis of malignant melanoma: A review and update. Hum. Pathol..

[74] Sundram U., Harvell J.D., Rouse R.V., Natkunam Y. (2003). Expression of the B-cell proliferation marker MUM1 by melanocytic lesions and comparison with S100, gp100 (HMB45), and MelanA. Mod. Pathol..

[75] Chen H.L., D’Mello S.R. (2010). Induction of neuronal cell death by paraneoplastic Ma1 antigen. J. Neurosci. Res..

[76] Jiang S.H., He P., Ma M.Z., Wang Y., Li R.K., Fang F., Fu Y., Tian G.A., Qin W.X., Zhang Z.G. (2014). PNMA1 promotes cell growth in human pancreatic ductal adenocarcinoma. Int. J. Clin. Exp. Pathol..

[77] Yang W., Lai Z., Li Y., Mu J., Yang M., Xie J., Xu J. (2019). Immune signature profiling identified prognostic factors for gastric cancer. Chin. J. Cancer. Res..

[78] Strand C., Enell J., Hedenfalk I., Ferno M. (2007). RNA quality in frozen breast cancer samples and the influence on gene expression analysis—A comparison of three evaluation methods using microcapillary electrophoresis traces. BMC Mol. Biol..

[79] Field M.G., Decatur C.L., Kurtenbach S., Gezgin G., van der Velden P.A., Jager M.J., Kozak K.N., Harbour J.W. (2016). PRAME as an Independent Biomarker for Metastasis in Uveal Melanoma. Clin. Cancer Res..

[80] Field M.G., Durante M.A., Decatur C.L., Tarlan B., Oelschlager K.M., Stone J.F., Kuznetsov J., Bowcock A.M., Kurtenbach S., Harbour J.W. (2016). Epigenetic reprogramming and aberrant expression of PRAME are associated with increased metastatic risk in Class 1 and Class 2 uveal melanomas. Oncotarget.

[81] Gezgin G., Luk S.J., Cao J., Dogrusoz M., van der Steen D.M., Hagedoorn R.S., Krijgsman D., van der Velden P.A., Field M.G., Luyten G.P.M. (2017). PRAME as a Potential Target for Immunotherapy in Metastatic Uveal Melanoma. JAMA Ophthalmol..

[82] Epping M.T., Wang L., Edel M.J., Carlee L., Hernandez M., Bernards R. (2005). The human tumor antigen PRAME is a dominant repressor of retinoic acid receptor signaling. Cell.

[83] Al-Khadairi G., Decock J. (2019). Cancer Testis Antigens and Immunotherapy: Where Do We Stand in the Targeting of PRAME?. Cancers.

[84] Mattheolabakis G., Milane L., Singh A., Amiji M.M. (2015). Hyaluronic acid targeting of CD44 for cancer therapy: From receptor biology to nanomedicine. J. Drug. Target..

[85] Chen C., Zhao S., Karnad A., Freeman J.W. (2018). The biology and role of CD44 in cancer progression: Therapeutic implications. J. Hematol. Oncol..

[86] Creyghton W.M., de Waard-Siebinga I., Danen E.H., Luyten G.P., van Muijen G.N., Jager M.J. (1995). Cytokine-mediated modulation of integrin, ICAM-1 and CD44 expression on human uveal melanoma cells in vitro. Melanoma Res..

[87] Danen E.H., ten Berge P.J., van Muijen G.N., Jager M.J., Ruiter D.J. (1996). Expression of CD44 and the pattern of CD44 alternative splicing in uveal melanoma. Melanoma Res..

[88] H Y.T.K. (2019). CD44-Targeting Nanocarriers for Cancer Treatment. Drug Deliv. System.

[89] Trzpis M., McLaughlin P.M., de Leij L.M., Harmsen M.C. (2007). Epithelial cell adhesion molecule: More than a carcinoma marker and adhesion molecule. Am. J. Pathol..

[90] Beutel J., Wegner J., Wegner R., Ziemssen F., Nassar K., Rohrbach J.M., Hilgers R.D., Luke M., Grisanti S. (2009). Possible implications of MCAM expression in metastasis and non-metastatic of primary uveal melanoma patients. Curr. Eye Res..

[91] Lai K., Sharma V., Jager M.J., Conway R.M., Madigan M.C. (2007). Expression and distribution of MUC18 in human uveal melanoma. Virchows Arch..

[92] Bande M.F., Santiago M., Muinelo-Romay L., Blanco M.J., Mera P., Capeans C., Pardo M., Pineiro A. (2015). Detection of circulating melanoma cells in choroidal melanocytic lesions. BMC Res. Notes.

[93] Crepin R., Gentien D., Duche A., Rapinat A., Reyes C., Nemati F., Massonnet G., Decaudin D., Djender S., Moutel S. (2017). Nanobodies against surface biomarkers enable the analysis of tumor genetic heterogeneity in uveal melanoma patient-derived xenografts. Pigment Cell Melanoma Res..

[94] Pardo M., Garcia A., Thomas B., Pineiro A., Akoulitchev A., Dwek R.A., Zitzmann N. (2006). The characterization of the invasion phenotype of uveal melanoma tumour cells shows the presence of MUC18 and HMG-1 metastasis markers and leads to the identification of DJ-1 as a potential serum biomarker. Int. J. Cancer.

[95] Colomb F., Wang W., Simpson D., Zafar M., Beynon R., Rhodes J.M., Yu L.G. (2017). Galectin-3 interacts with the cell-surface glycoprotein CD146 (MCAM, MUC18) and induces secretion of metastasis-promoting cytokines from vascular endothelial cells. J. Biol. Chem..

[96] Duckworth C.A., Guimond S.E., Sindrewicz P., Hughes A.J., French N.S., Lian L.Y., Yates E.A., Pritchard D.M., Rhodes J.M., Turnbull J.E. (2015). Chemically modified, non-anticoagulant heparin derivatives are potent galectin-3 binding inhibitors and inhibit circulating galectin-3-promoted metastasis. Oncotarget.

[97] Tsujimoto Y., Finger L.R., Yunis J., Nowell P.C., Croce C.M. (1984). Cloning of the chromosome breakpoint of neoplastic B cells with the t(14;18) chromosome translocation. Science.

[98] Mooy C.M., Luyten G.P., de Jong P.T., Luider T.M., Stijnen T., van de Ham F., van Vroonhoven C.C., Bosman F.T. (1995). Immunohistochemical and prognostic analysis of apoptosis and proliferation in uveal melanoma. Am. J. Pathol..

[99] Coupland S.E., Bechrakis N., Schuler A., Anagnostopoulos I., Hummel M., Bornfeld N., Stein H. (1998). Expression patterns of cyclin D1 and related proteins regulating G1-S phase transition in uveal melanoma and retinoblastoma. Br. J. Ophthalmol..

[100] Chana J.S., Wilson G.D., Cree I.A., Alexander R.A., Myatt N., Neale M., Foss A.J., Hungerford J.L. (1999). c-myc, p53, and Bcl-2 expression and clinical outcome in uveal melanoma. Br. J. Ophthalmol..

[101] Sulkowska M., Famulski W., Bakunowicz-Lazarczyk A., Chyczewski L., Sulkowski S. (2001). Bcl-2 expression in primary uveal melanoma. Tumori J..

[102] He M., Chaurushiya M.S., Webster J.D., Kummerfeld S., Reja R., Chaudhuri S., Chen Y.J., Modrusan Z., Haley B., Dugger D.L. (2019). Intrinsic apoptosis shapes the tumor spectrum linked to inactivation of the deubiquitinase BAP1. Science.

[103] Nemati F., de Montrion C., Lang G., Kraus-Berthier L., Carita G., Sastre-Garau X., Berniard A., Vallerand D., Geneste O., de Plater L. (2014). Targeting Bcl-2/Bcl-XL induces antitumor activity in uveal melanoma patient-derived xenografts. PLoS ONE.

[104] Wang J., Jia R., Zhang Y., Xu X., Song X., Zhou Y., Zhang H., Ge S., Fan X. (2014). The role of Bax and Bcl-2 in gemcitabine-mediated cytotoxicity in uveal melanoma cells. Tumour Biol..

[105] Bellini L., Strub T., Habel N., Pandiani C., Marchetti S., Martel A., Baillif S., Bailly-Maitre B., Gual P., Ballotti R. (2020). Endoplasmic reticulum stress mediates resistance to BCL-2 inhibitor in uveal melanoma cells. Cell Death Discov..

[106] Decaudin D., Frisch Dit Leitz E., Nemati F., Tarin M., Naguez A., Zerara M., Marande B., Vivet-Noguer R., Halilovic E., Fabre C. (2020). Preclinical evaluation of drug combinations identifies co-inhibition of Bcl-2/XL/W and MDM2 as a potential therapy in uveal melanoma. Eur. J. Cancer.

[107] Harazono Y., Kho D.H., Balan V., Nakajima K., Zhang T., Hogan V., Raz A. (2014). Galectin-3 leads to attenuation of apoptosis through Bax heterodimerization in human thyroid carcinoma cells. Oncotarget.

[108] All-Ericsson C., Girnita L., Seregard S., Bartolazzi A., Jager M.J., Larsson O. (2002). Insulin-like growth factor-1 receptor in uveal melanoma: A predictor for metastatic disease and a potential therapeutic target. Investig. Ophthalmol. Vis. Sci..

[109] Topcu-Yilmaz P., Kiratli H., Saglam A., Soylemezoglu F., Hascelik G. (2010). Correlation of clinicopathological parameters with HGF, c-Met, EGFR, and IGF-1R expression in uveal melanoma. Melanoma Res..

[110] Al-Jamal R.T., Kivela T. (2011). Prognostic associations of insulin-like growth factor-1 receptor in primary uveal melanoma. Can. J. Ophthalmol..

[111] Wu X., Zhou J., Rogers A.M., Janne P.A., Benedettini E., Loda M., Hodi F.S. (2012). c-Met, epidermal growth factor receptor, and insulin-like growth factor-1 receptor are important for growth in uveal melanoma and independently contribute to migration and metastatic potential. Melanoma Res..

[112] Yoshida M., Selvan S., McCue P.A., DeAngelis T., Baserga R., Fujii A., Rui H., Mastrangelo M.J., Sato T. (2014). Expression of insulin-like growth factor-1 receptor in metastatic uveal melanoma and implications for potential autocrine and paracrine tumor cell growth. Pigment Cell Melanoma Res..

[113] Xie X., Xie S., Xie C., Fang Y., Li Z., Wang R., Jiang W. (2019). Pristimerin attenuates cell proliferation of uveal melanoma cells by inhibiting insulin-like growth factor-1 receptor and its downstream pathways. J. Cell Mol. Med..

[114] Faiao-Flores F., Emmons M.F., Durante M.A., Kinose F., Saha B., Fang B., Koomen J.M., Chellappan S.P., Maria-Engler S.S., Rix U. (2019). HDAC Inhibition Enhances the In Vivo Efficacy of MEK Inhibitor Therapy in Uveal Melanoma. Clin. Cancer. Res..

[115] Shain A.H., Bagger M.M., Yu R., Chang D., Liu S., Vemula S., Weier J.F., Wadt K., Heegaard S., Bastian B.C. (2019). The genetic evolution of metastatic uveal melanoma. Nat. Genet..

[116] Dopierala J., Damato B.E., Lake S.L., Taktak A.F., Coupland S.E. (2010). Genetic heterogeneity in uveal melanoma assessed by multiplex ligation-dependent probe amplification. Investig. Ophthalmol. Vis. Sci..

[117] Damato B., Dopierala J.A., Coupland S.E. (2010). Genotypic profiling of 452 choroidal melanomas with multiplex ligation-dependent probe amplification. Clin. Cancer Res..

[118] Galon J., Costes A., Sanchez-Cabo F., Kirilovsky A., Mlecnik B., Lagorce-Pages C., Tosolini M., Camus M., Berger A., Wind P. (2006). Type, density, and location of immune cells within human colorectal tumors predict clinical outcome. Science.

[119] Galon J., Mlecnik B., Bindea G., Angell H.K., Berger A., Lagorce C., Lugli A., Zlobec I., Hartmann A., Bifulco C. (2014). Towards the introduction of the ’Immunoscore’ in the classification of malignant tumours. J. Pathol..

[120] Galon J., Bruni D. (2019). Approaches to treat immune hot, altered and cold tumours with combination immunotherapies. Nat. Rev. Drug. Discov..

[121] Farquhar N., Thornton S., Coupland S.E., Coulson J.M., Sacco J.J., Krishna Y., Heimann H., Taktak A., Cebulla C.M., Abdel-Rahman M.H. (2018). Patterns of BAP1 protein expression provide insights into prognostic significance and the biology of uveal melanoma. J. Pathol. Clin. Res..

