# Supplementary material for: Transcriptome Profiling Reveals New Insights into the Immune Microenvironment and Upregulation of Novel Biomarkers in Metastatic Uveal Melanoma
Source: Cancers (Basel). 2020 Sep 30;12(10):2832. doi: 10.3390/cancers12102832 (PMC7650807; doi:10.3390/cancers12102832)
Supplement: Supplementary file 1 [file cancers-12-02832-s001.zip › Suppl figs/Figure S1.pptx]

## Slide 1
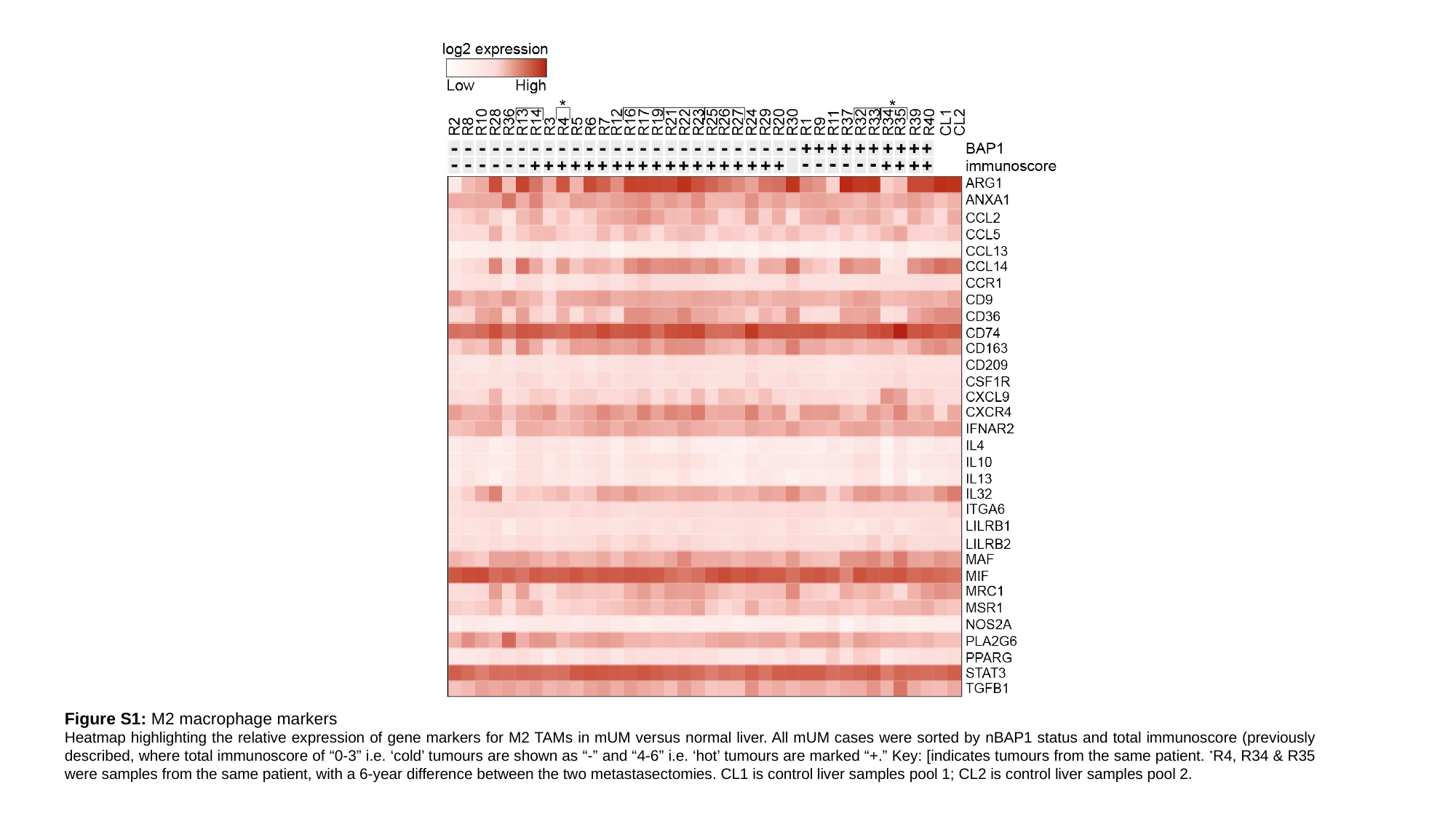

Figure S1: M2 macrophage markers
Heatmap highlighting the relative expression of gene markers for M2 TAMs in mUM versus normal liver. All mUM cases were sorted by nBAP1 status and total immunoscore (previously described, where total immunoscore of “0-3” i.e. ‘cold’ tumours are shown as “-” and “4-6” i.e. ‘hot’ tumours are marked “+.” Key: [indicates tumours from the same patient. *R4, R34 & R35 were samples from the same patient, with a 6-year difference between the two metastasectomies. CL1 is control liver samples pool 1; CL2 is control liver samples pool 2.
