# Supplementary material for: Transcriptome Profiling Reveals New Insights into the Immune Microenvironment and Upregulation of Novel Biomarkers in Metastatic Uveal Melanoma
Source: Cancers (Basel). 2020 Sep 30;12(10):2832. doi: 10.3390/cancers12102832 (PMC7650807; doi:10.3390/cancers12102832)
Supplement: Supplementary file 1 [file cancers-12-02832-s001.zip › Suppl figs/Figure S2.pptx]

## Slide 1
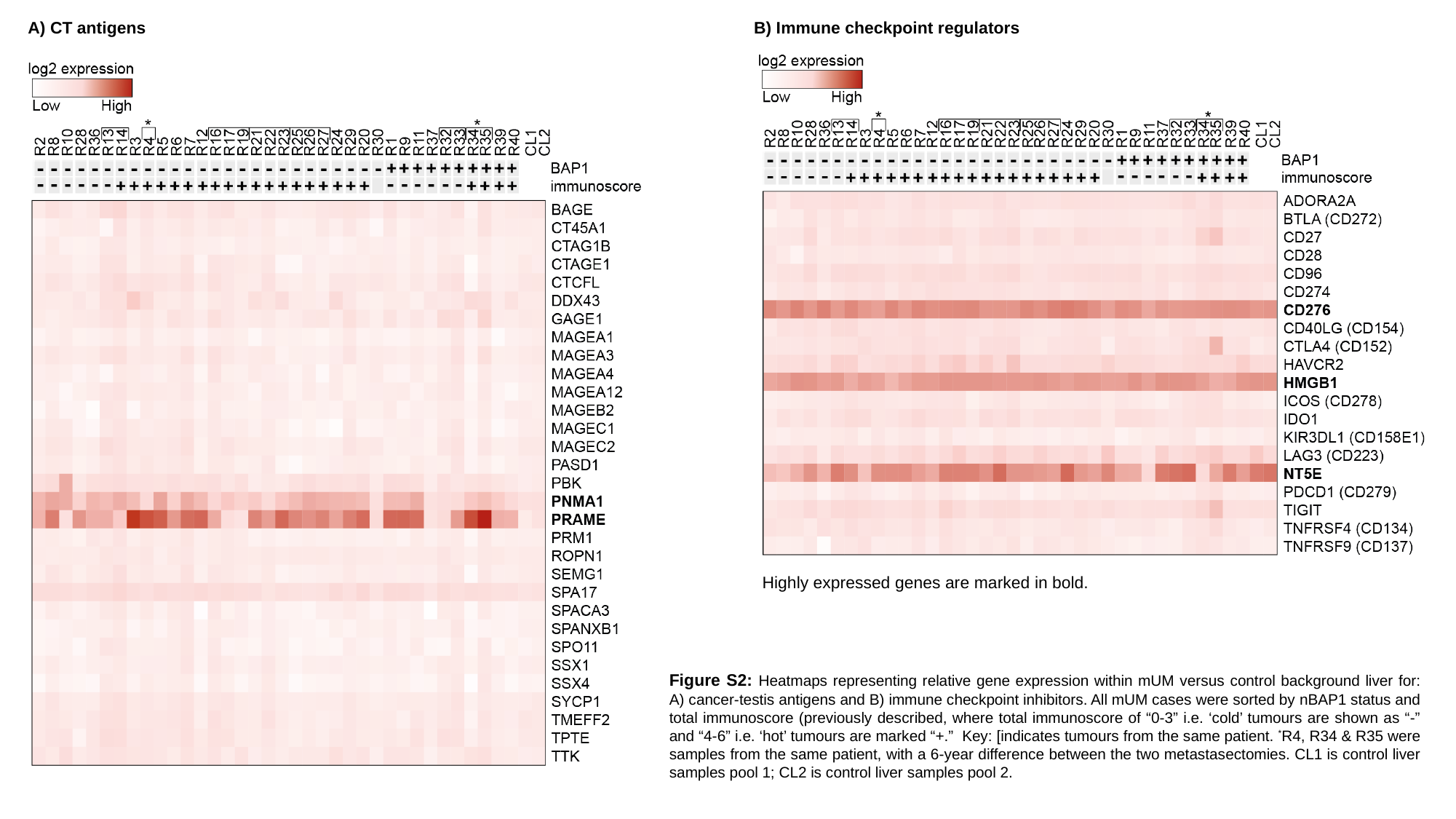

A) CT antigens
B) Immune checkpoint regulators
Highly expressed genes are marked in bold.
Figure S2: Heatmaps representing relative gene expression within mUM versus control background liver for: A) cancer-testis antigens and B) immune checkpoint inhibitors. All mUM cases were sorted by nBAP1 status and total immunoscore (previously described, where total immunoscore of “0-3” i.e. ‘cold’ tumours are shown as “-” and “4-6” i.e. ‘hot’ tumours are marked “+.” Key: [indicates tumours from the same patient. *R4, R34 & R35 were samples from the same patient, with a 6-year difference between the two metastasectomies. CL1 is control liver samples pool 1; CL2 is control liver samples pool 2.
