# Supplementary material for: Transcriptome Profiling Reveals New Insights into the Immune Microenvironment and Upregulation of Novel Biomarkers in Metastatic Uveal Melanoma
Source: Cancers (Basel). 2020 Sep 30;12(10):2832. doi: 10.3390/cancers12102832 (PMC7650807; doi:10.3390/cancers12102832)
Supplement: Supplementary file 1 [file cancers-12-02832-s001.zip › Suppl figs/Figure S3.pptx]

## Slide 1
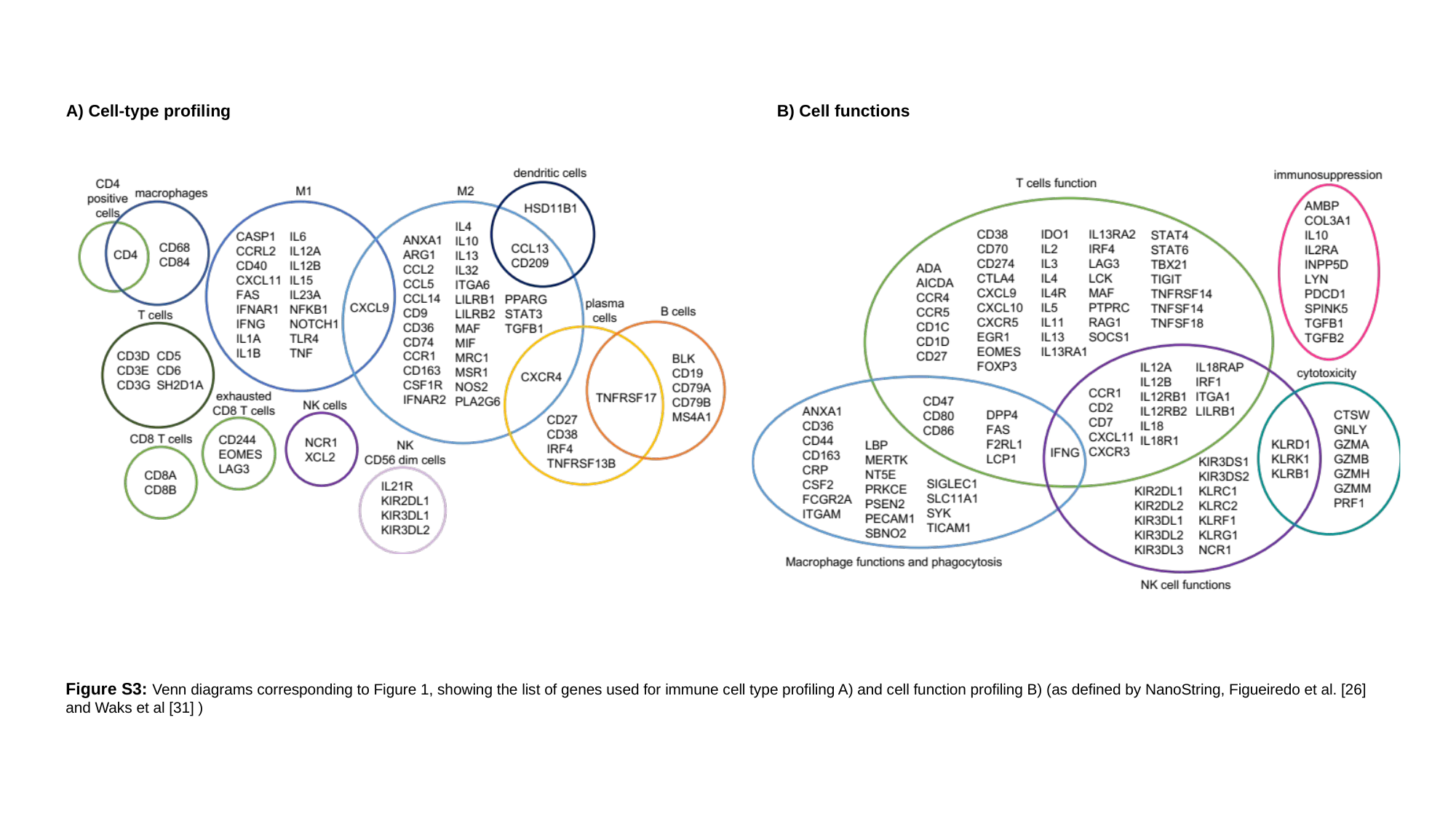

A) Cell-type profiling
B) Cell functions
Figure S3: Venn diagrams corresponding to Figure 1, showing the list of genes used for immune cell type profiling A) and cell function profiling B) (as defined by NanoString, Figueiredo et al. [26] and Waks et al [31] )
