# Supplementary material for: Transcriptome Profiling Reveals New Insights into the Immune Microenvironment and Upregulation of Novel Biomarkers in Metastatic Uveal Melanoma
Source: Cancers (Basel). 2020 Sep 30;12(10):2832. doi: 10.3390/cancers12102832 (PMC7650807; doi:10.3390/cancers12102832)
Supplement: Supplementary file 1 [file cancers-12-02832-s001.zip › Suppl figs/Figure S4.pptx]

## Slide 1
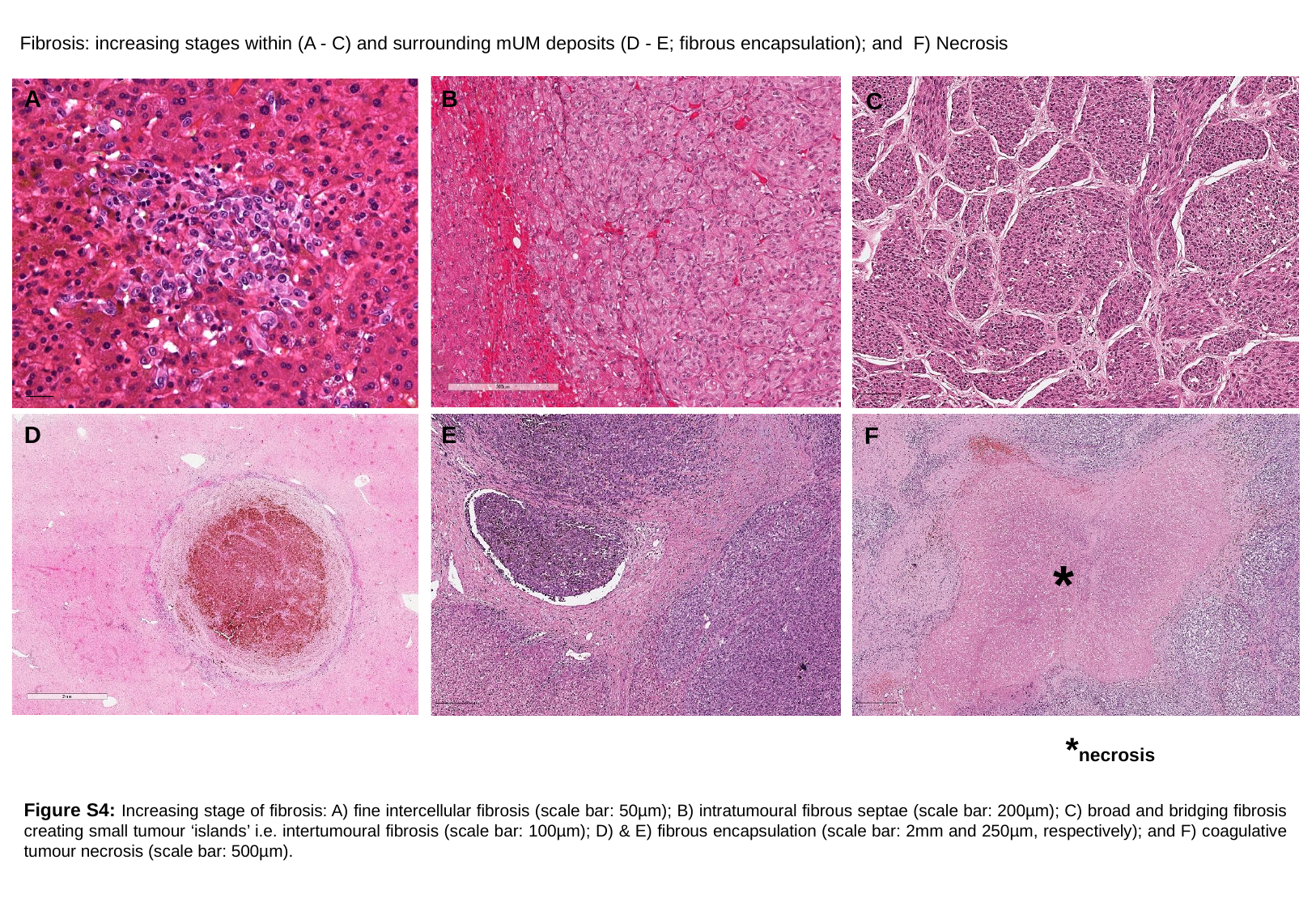

Fibrosis: increasing stages within (A - C) and surrounding mUM deposits (D - E; fibrous encapsulation); and F) Necrosis
A
B
C
E
D
F
*
*necrosis
Figure S4: Increasing stage of fibrosis: A) fine intercellular fibrosis (scale bar: 50µm); B) intratumoural fibrous septae (scale bar: 200µm); C) broad and bridging fibrosis creating small tumour ‘islands’ i.e. intertumoural fibrosis (scale bar: 100µm); D) & E) fibrous encapsulation (scale bar: 2mm and 250µm, respectively); and F) coagulative tumour necrosis (scale bar: 500µm).
