# Supplementary material for: Transcriptome Profiling Reveals New Insights into the Immune Microenvironment and Upregulation of Novel Biomarkers in Metastatic Uveal Melanoma
Source: Cancers (Basel). 2020 Sep 30;12(10):2832. doi: 10.3390/cancers12102832 (PMC7650807; doi:10.3390/cancers12102832)
Supplement: Supplementary file 1 [file cancers-12-02832-s001.zip › Suppl figs/Figure S5.pptx]

## Slide 1
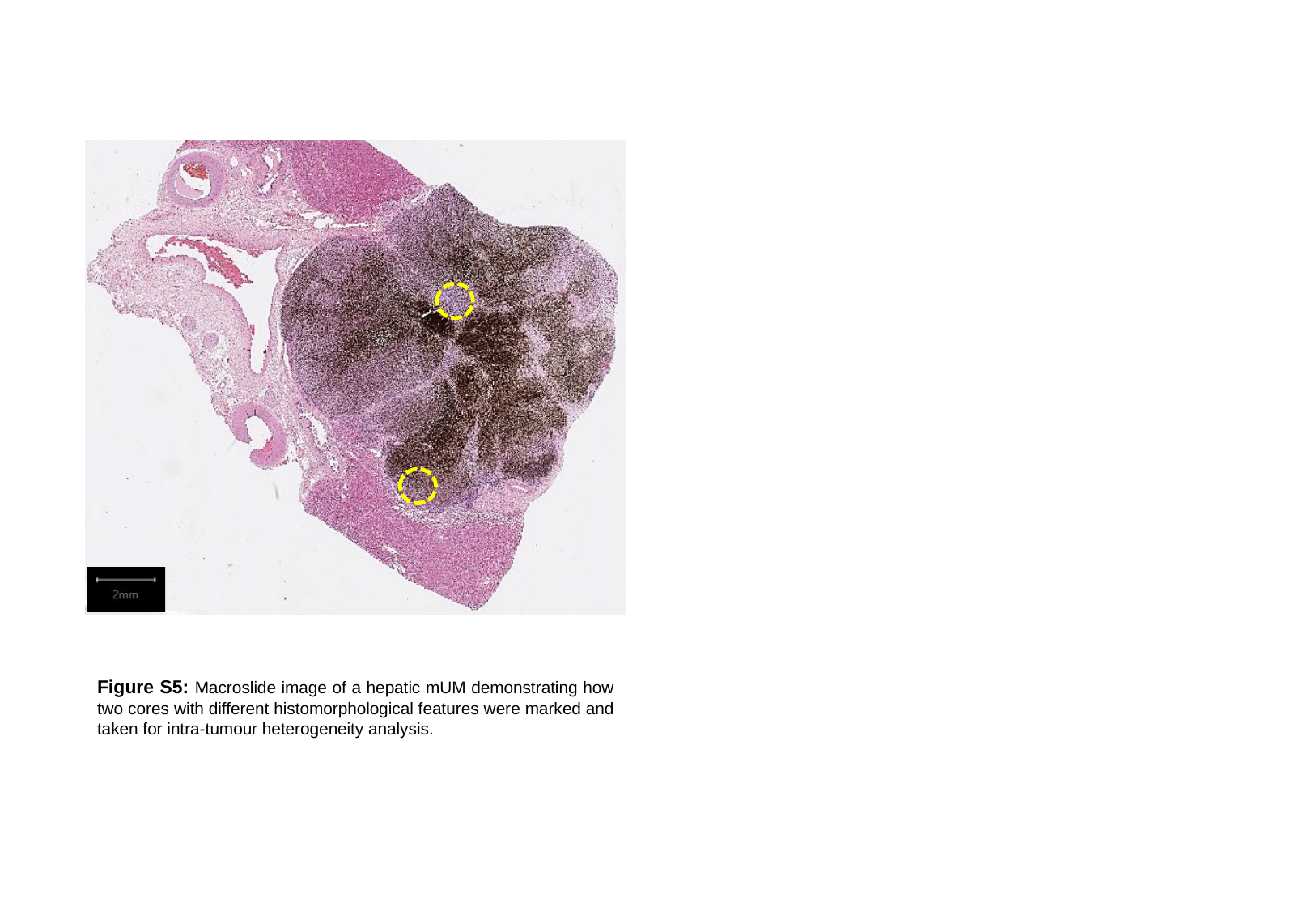

Figure S5: Macroslide image of a hepatic mUM demonstrating how two cores with different histomorphological features were marked and taken for intra-tumour heterogeneity analysis.
