# Supplementary material for: Transcriptome Profiling Reveals New Insights into the Immune Microenvironment and Upregulation of Novel Biomarkers in Metastatic Uveal Melanoma
Source: Cancers (Basel). 2020 Sep 30;12(10):2832. doi: 10.3390/cancers12102832 (PMC7650807; doi:10.3390/cancers12102832)
Supplement: Supplementary file 1 [file cancers-12-02832-s001.zip › Suppl tables/Table S1.docx]

**Table S1:** Clinical and histomorphological information for all the hepatic mUM samples.

| **mUM NanoString ID** | **Age PMx** | **Gender** | **Chr3 Status** | **Growth Pattern** | **H&E** | **Fibrosis Encapsulation** | **nBAP1** | **Total Immunoscore** |
| --- | --- | --- | --- | --- | --- | --- | --- | --- |
| R1 | 66 | F |  | nodular | spindle | Y | Pos | Low |
| R2 |  | F |  | infiltrative | epithelioid, discohesive, patchy fibrosis, necrosis | N | Neg | Low |
| R3 | 63 | M | L | nodular | epithelioid | Y | Neg | High |
| R4^$^ | 54 | M | L | nodular | epithelioid, heavy pigment | Y | Neg | High |
| R5 | 57 | F | L | nodular | epithelioid | Y | Neg | High |
| R6 | 39 | F | L | nodular | epithelioid, partial pigmented | Y | Neg | High |
| R7 | 56 | F | L | nodular | mixed, pigmented, necrosis | Y | Neg | High |
| R8 | 68 | F | L | infiltrative | mixed, partial pigmented, patchy fibrosis | N | Neg | Low |
| R9 | 38 | F |  | nodular | mixed, necrosis, patchy fibrosis | Y | Pos | Low |
| R10 | 64 | F | L | infiltrative | epithelioid, necrosis, patchy fibrosis | N | Neg | Low |
| R11 | 54 | M |  | nodular | spindle, partial pigmented, fibrosis, necrosis++ | Y | Pos | Low |
| R12 | 64 | M | L | nodular | epithelioid, patchy necrosis & fibrosis | N | Neg | High |
| R13 | 66 | M | L | nodular | epithelioid, patchy fibrosis | N | Neg | Low |
| R14 | 66 | M | L | nodular | epithelioid, patchy fibrosis | Y | Neg | High |
| R15* | 66 | M | L | nodular | epithelioid, patchy fibrosis | Y | Neg | High |
| R16 | 53 | F | L | nodular | epithelioid, patchy necrosis & fibrosis | Y | Neg | High |
| R17 | 53 | F | L | nodular | epithelioid, patchy necrosis & fibrosis | Y | Neg | High |
| R18* | 53 | F | L | nodular | epithelioid, patchy fibrosis | Y | Neg | High |
| R19 | 53 | F | L | nodular | epithelioid, patchy fibrosis | Y | Neg | High |
| R20 | 38 | F |  | infiltrative | epithelioid partial pigmented | N | Neg | High |
| R21 *(R41)* | 62 | M | L | nodular | epithelioid heavy pigment | Y | Neg | High |
| R22 | 62 | M | L | nodular | epithelioid heavy pigment | Y | Neg | High |
| R23 | 62 | M | L | nodular | epithelioid pigmented, patchy fibrosis | Y | Neg | High |
| R24 | 67 | M | L | infiltrative | epithelioid, patchy fibrosis, necrosis | N | Neg | High |
| R25 *(R42, R43)* | 54 | M | L | nodular | epithelioid, partial pigmented, patchy fibrosis | Y | Neg | High |
| R26 | 54 | M | L | nodular | epithelioid, partial pigmented, discohesive, patchy fibrosis | Y | Neg | High |
| R27 | 54 | M | L | nodular | epithelioid, pigmented, patchy fibrosis | Y | Neg | High |
| R28 | 47 | M | L | infiltrative | epithelioid, partial pigmented, patchy fibrosis | N | Neg | Low |
| R29 | 46 | M | L | infiltrative | epithelioid, partial pigmented | N | Neg | High |
| R30 | 75 | M | L | infiltrative | epithelioid | N | Neg |  |
| R31* | 45 | M | N | nodular | mixed main spindle, patchy necrosis | N | Pos | Low |
| R32 | 45 | M | N | nodular | mixed, main spindle | N | Pos | Low |
| R33 | 45 | M | N | nodular | mixed | N | Pos | Low |
| R34^$^ *(R44)* | 54 | M | L | nodular | mixed, patchy fibrosis & necrosis | N | Pos | High |
| R35^$^ | 54 | M | L | nodular | mixed, partial pigment, patchy fibrosis & necrosis | N | Pos | High |
| R36 | 32 | M | L | nodular | epithelioid | Y | Neg | Low |
| R37 | 38 | M | L | nodular | epithelioid, patchy fibrosis, necrosis | N | Pos | Low |
| R38* | 78 | F | L | nodular | epithelioid, partial pigmented | N | Neg | Low |
| R39 | 67 | F | L | infiltrative | epithelioid, partial pigmented, patchy fibrosis, necrosis | N | Pos | High |
| R40 *(R45)* | 46 | M | N | nodular | epithelioid, heavy pigment, patchy fibrosis | N | Pos | High |

Key: *Excluded samples from mUM versus control liver analyses. [ indicates tumours from the same patient. ^$^R4, R34 & R35 were samples from the same patient. *(R41-45)* were mUM from different liver segments from the same patients. These samples were only used for the “intersegmental” and “intratumoural” analyses and not in the main mUM versus control study arm. Chromosome status of the primary UM was missing in 5 patients (1x block missing from archive, 1x primary management elsewhere therefore no primary tumour block, 2x had proton beam therapy as primary management and therefore the primary tumour was not sampled, 1x 25G transretinal biopsy which was predominantly fluid with scattered UM cells).
