# Supplementary material for: Transcriptome Profiling Reveals New Insights into the Immune Microenvironment and Upregulation of Novel Biomarkers in Metastatic Uveal Melanoma
Source: Cancers (Basel). 2020 Sep 30;12(10):2832. doi: 10.3390/cancers12102832 (PMC7650807; doi:10.3390/cancers12102832)
Supplement: Supplementary file 1 [file cancers-12-02832-s001.zip › Suppl tables/Table S3.pptx]

## Slide 1
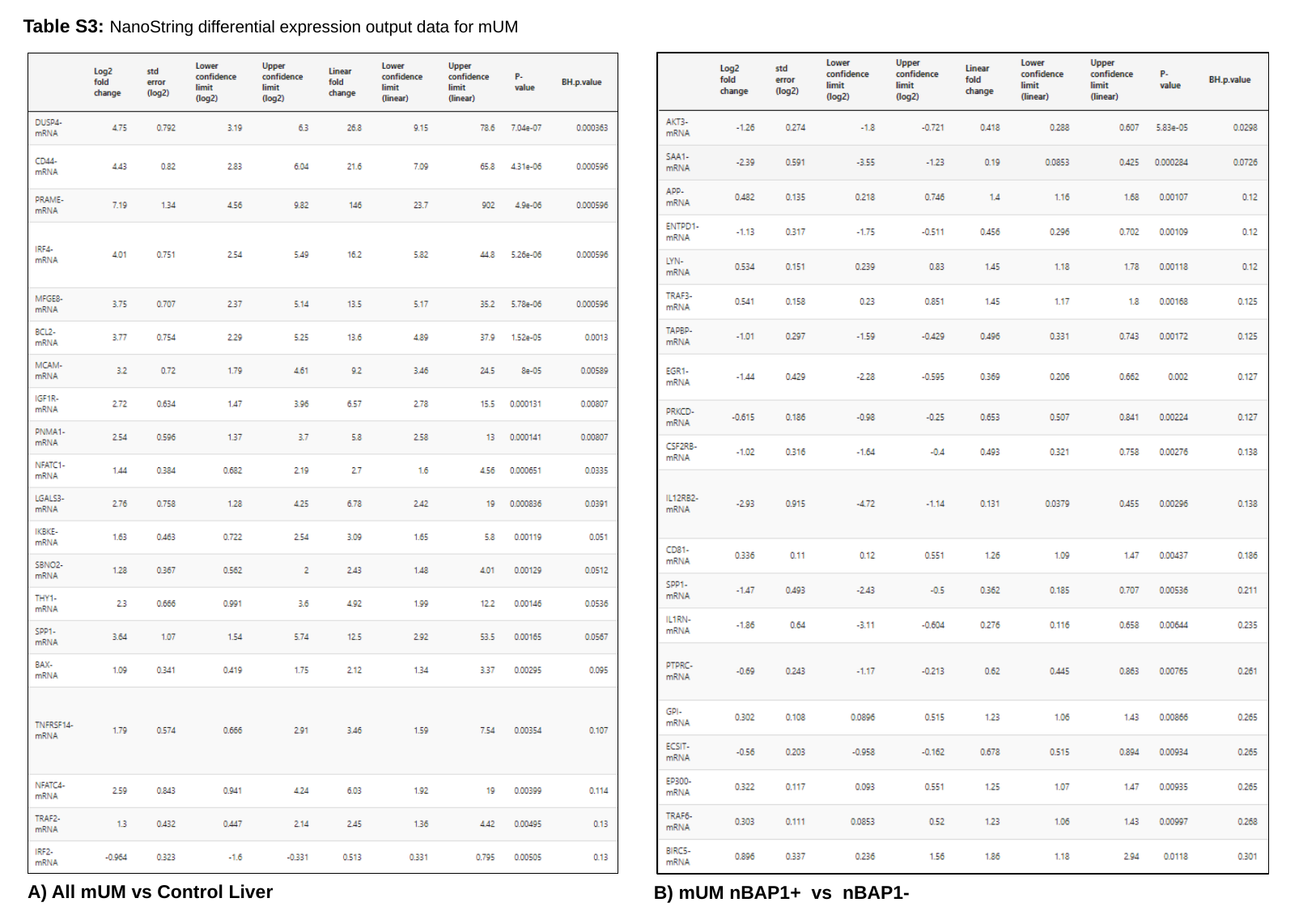

Table S3: NanoString differential expression output data for mUM
A) All mUM vs Control Liver
B) mUM nBAP1+ vs nBAP1-
