# Supplementary material for: Transcriptome Profiling Reveals New Insights into the Immune Microenvironment and Upregulation of Novel Biomarkers in Metastatic Uveal Melanoma
Source: Cancers (Basel). 2020 Sep 30;12(10):2832. doi: 10.3390/cancers12102832 (PMC7650807; doi:10.3390/cancers12102832)
Supplement: Supplementary file 1 [file cancers-12-02832-s001.zip › Suppl tables/Table S4.docx]

**Table S4:** Summary of IHC results.

|  | | **DUSP4** | **CD44** | **PRAME** | **IRF4** | **BCL2** | **CD146** | **IGF1R** | **LGALS3** |
| --- | --- | --- | --- | --- | --- | --- | --- | --- | --- |
| Cases with tumour cells positive | | 18/19 | 19/19 | 2/20 | 1/19 | 19/19 | 19/19 | 18/20 | 2/19 |
| % of positive cells within tumour | 75-100% | 18/19 | 19/19 | 2/20 | 0/19 | 19/19 | 16/19 | 15/20 | 2/19 |
|  | 50-74% | 0/19 | 0/19 | 0/20 | 0/19 | 0/19 | 1/19 | 3/20 | 0/19 |
|  | 25-49% | 0/19 | 0/19 | 0/20 | 0/19 | 0/19 | 1/19 | 1/20 | 0/19 |
|  | 1-24% | 0/19 | 0/19 | 0/20 | 1/19 | 0/19 | 1/19 | 1/20 | 0/19 |
| Signal localisation in tumour cells | | N, C,  N and C | C, M | N | N | C | C, M | C | C |
| Other positive cells | | Hepatocytes (scattered nuclear stain) | MQ | na | Plasma cells | na | MQ | MQ | MQ |

**Key**: Localisation C – cytoplasmic; N – nuclear; M – membrane; MQ – macrophages; na – non-applicable.
