# Supplementary material for: Transcriptome Profiling Reveals New Insights into the Immune Microenvironment and Upregulation of Novel Biomarkers in Metastatic Uveal Melanoma
Source: Cancers (Basel). 2020 Sep 30;12(10):2832. doi: 10.3390/cancers12102832 (PMC7650807; doi:10.3390/cancers12102832)
Supplement: Supplementary file 1 [file cancers-12-02832-s001.zip › Suppl tables/Table S5.docx]

**Table S5:** Details of the antibodies used for IHC

| **Antibody** | **Full name; Gene location** | **Supplier** | **Dilution** | **Positive control** |
| --- | --- | --- | --- | --- |
| DUSP4 | Dual specificity phosphatase 4; chr8p12 | Abcam | 1:100 | Brain |
| PRAME | Preferentially expressed antigen in melanoma; chr22q11.22 | Abcam | 1:500 | Testis |
| CD44 | Cluster of Differentiation 44; chr11p13 | DAKO | 1:20 | Tonsil |
| IRF4 | Interferon Regulatory Factor 4 (also called MUM1); chr6p25.3 | DAKO | 1:100 | Colon and lymphoid tissue |
| BCL-2 | B-cell lymphoma 2; chr18q21.33 | DAKO | 1:500 | Tonsil |
| LGALS3 | Galectin 3; chr14q22.3 | Sigma | 1:100 | Skin |
| MFGE8 | Milk fat globule-EGF factor 8 (also called lactadherin); chr 15q26.1 | Abcam | 1:200 | Breast |
| CD146/MCAM/MUC1 | Melanoma Cell Adhesion Molecule (also termed MUC1); chr 11q23.3 | Abcam | 1:250 | Pancreas |
| ITGB4 | Integrin Subunit Beta 4 (also called CD104) chr 17q25.1 | Abcam | 1:250 | Colon |
| IL2RA | Interleukin 2 Receptor Subunit Alpha (also called CD25), chr 10p15.1 | Sigma | 1:200 | Tonsil |
| IGF1 | Insulin Like Growth Factor 1 chr 12q23.2 | Abcam | 1:250 | Prostate |
| CCL4 | C-C Motif Chemokine Ligand 4 chr 17q12 | Abcam | 1:50 | Kidney |
| AKT3 | AKT Serine/Threonine Kinase 3 chr 1q43-q44 | Abcam | 1:200 | Kidney |
